# Protein posttranslational modifications in metabolic diseases: basic concepts and targeted therapies

**DOI:** 10.1002/mco2.752

**Published:** 2024-09-30

**Authors:** Yunuo Yang, Jiaxuan Wu, Wenjun Zhou, Guang Ji, Yanqi Dang

**Affiliations:** ^1^ Institute of Digestive Diseases China‐Canada Center of Research for Digestive Diseases (ccCRDD) Shanghai University of Traditional Chinese Medicine Shanghai China; ^2^ State Key Laboratory of Integration and Innovation of Classic Formula and Modern Chinese Medicine (Shanghai University of Traditional Chinese Medicine) Shanghai China

**Keywords:** diabetes mellitus, hyperlipidemia, nonalcoholic fatty liver disease, obesity, protein posttranslational modification

## Abstract

Metabolism‐related diseases, including diabetes mellitus, obesity, hyperlipidemia, and nonalcoholic fatty liver disease, are becoming increasingly prevalent, thereby posing significant threats to human health and longevity. Proteins, as the primary mediators of biological activities, undergo various posttranslational modifications (PTMs), including phosphorylation, ubiquitination, acetylation, methylation, and SUMOylation, among others, which substantially diversify their functions. These modifications are crucial in the physiological and pathological processes associated with metabolic disorders. Despite advancements in the field, there remains a deficiency in contemporary summaries addressing how these modifications influence processes of metabolic disease. This review aims to systematically elucidate the mechanisms through which PTM of proteins impact the progression of metabolic diseases, including diabetes, obesity, hyperlipidemia, and nonalcoholic fatty liver disease. Additionally, the limitations of the current body of research are critically assessed. Leveraging PTMs of proteins provides novel insights and therapeutic targets for the prevention and treatment of metabolic disorders. Numerous drugs designed to target these modifications are currently in preclinical or clinical trials. This review also provides a comprehensive summary. By elucidating the intricate interplay between PTMs and metabolic pathways, this study advances understanding of the molecular mechanisms underlying metabolic dysfunction, thereby facilitating the development of more precise and effective disease management strategies.

## Graphical abstract

Posttranslational modifications of proteins are involved in the development of metabolism‐related diseases such as diabetes mellitus, obesity, hyperlipidemia, and nonalcoholic fatty liver disease. PTMs mediates the development of diabetes mellitus by affecting insulin homeostasis, glucose metabolism, diabetic complications, gluconeogenesis, and β‐cell function. PTMs is involved in obesity by affecting autophagy, FA metabolism, lipid accumulation, and insulin resistance. PTM influences cholesterol metabolism, FA metabolism, and lipid accumulation in the development of hyperlipidemia. PTMs is involved in the development of NAFLD by affecting FA oxidation, autophagy, lactate accumulation, insulin signaling pathway and inflammation.

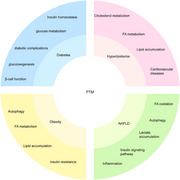

## 1 INTRODUCTION

With socio‐economic development, human habits are changing. Concurrently, an aging population exacerbates the incidence of metabolic diseases marked by dysregulated metabolism, notably including diabetes mellitus (DM), obesity, hyperlipidemia, and nonalcoholic fatty liver disease (NAFLD), posing significant threats to human health and longevity.^1^, ^2^

DM is a metabolic disorder distinguished by elevated blood glucose levels. Globally, its prevalence among adults is estimated at 9%, with type 2 diabetes mellitus (T2DM) comprising 90% of cases.^3^, ^4^ In 2017, approximately 425 million individuals were affected by DM, a figure projected to rise to 642 million by 2040.^5^ Obesity, characterized by an abnormal accumulation of fat detrimental to health, is typically diagnosed when an individual's body mass index reaches or exceeds 30, as defined by the World Health Organization. As of 2016, over 1.9 billion adults worldwide, along with more than 340 million children and adolescents aged from 5 to 19 years, were classified as overweight or obese,^6^, ^7^, ^8^, ^9^ with the number of obese individuals having tripled since 1975. Hyperlipidemia is characterized by elevated levels of lipids, including cholesterol, lipoproteins. In the United States, over 50% of adults exhibit elevated low‐density lipoprotein (LDL) levels.^10^ NAFLD, a clinicopathological syndrome, is typified by excessive accumulation of intracellular fat within hepatocytes, often accompanied by obesity, insulin resistance or T2DM, and dyslipidemia. Globally, the prevalence of NAFLD is approximately 25%,^11^, ^12^ with rates exceeding 30% in South America.^13^, ^14^ As one of the foremost chronic liver diseases worldwide, NAFLD imposes a substantial burden on healthcare systems.^15^ These metabolic diseases are independent of each other, but also interrelated and interact with each other. The combination of two or more metabolism‐related diseases often occurs in clinical practice. NAFLD is closely related to insulin resistance and often coexists with T2DM, and studies have shown that NAFLD can increase the risk of T2DM.^16^ A meta‐analysis of 80 studies suggests T2DM promotes disease progression in NAFLD.^17^ Quek et al.^18^ analyzed that the prevalence of NAFLD was 75.7% in the obese population. T2DM is a major risk factor for the development of cardiovascular disease, and obesity (especially the accumulation of visceral fat) also increases the risk of T2DM and cardiovascular disease by affecting cardiac metabolism.^19^ The increasing prevalence of metabolic diseases poses significant risks and challenges to human health and societal healthcare systems. Immediate and effective measures are essential for the prevention and treatment of metabolic disorders.

Epigenetics encompasses heritable changes in gene function, with the epigenome comprising DNA methylation, histone modifications, and noncoding RNAs.^20^ Among them, proteins serve as vital components in the functioning of living organisms, acting as primary agents for life processes. The human genome encodes approximately 20,000 genes, which undergo transcription and translation to produce proteins. Human metabolism is regulated by a variety of proteins, and protein function is affected by a variety of factors. Recent evidence suggests that posttranslational modifications (PTMs) of proteins affect glycolipid metabolism and are an important part of the development of metabolism‐related diseases.^21^ PTMs represents a crucial mechanism wherein proteins undergo covalent alterations, involving the addition of functional groups to amino acid residues or enzymatic cleavage. PTMs significantly enhance the functional diversity of proteins, leading to an exponential increase in protein variants, thereby contributing to the intricacies of biological activities. Current advancements in science and technology have identified over 600 types of PTMs. Numerous studies have demonstrated that PTMs are crucial in the onset and progression of various diseases. Among the diverse array of PTMs, phosphorylation, ubiquitination, acetylation, methylation, palmitoylation, succinylation, crotonylation, lactylation, malonylation, SUMOylation, S‐sulfhydration, glycosylation, and others are intimately associated with metabolic homeostasis. Consequently, this discussion will concentrate on the roles of these specific modifications in metabolic diseases.^22^, ^23^ Understanding the pathogenesis of metabolic diseases from the perspective of PTMs may provide new perspectives on the prevention and treatment of the diseases.

This review systematically elucidates the relationship between PTMs of proteins and metabolic disease processes, including DM, obesity, hyperlipidemia, and NAFLD, as well as therapeutic agents targeting PTMs. Additionally, we critically assess the limitations of current studies, providing new insights into the development of novel diagnostic biomarkers and therapeutic interventions for metabolic diseases.

## 2 PROTEIN PTMs

Precursor proteins undergo covalent processing, specifically PTMs, to transform into mature proteins with appropriate physiological functions. These PTMs have significantly expanded the diversity of proteins and added complexity to biological processes. Research has identified over 600 distinct types of protein PTMs. Among these, the most extensively studied modifications, particularly those closely associated with metabolism‐related diseases, include the following.

### 2.1 Phosphorylation

Phosphorylation represents one of the most prevalent types of covalent modifications in proteins. This biochemical process involves the transfer of a phosphoryl group from the γ‐position of adenosine triphosphate (ATP) or guanosine triphosphate to specific amino acid residues on the substrate protein.^24^ Phosphorylation of proteins leads to the incorporation of a net negative charge onto the amino acid residues of the phosphate group receptor. The regulation of histone modifications involves three principal classes of proteins: Writers, Erasers, and Readers. In the context of phosphorylation modifications, protein kinases (Writers) are responsible for the addition of phosphoryl groups, phosphorylation‐binding proteins (Readers) recognize and interact with the phosphorylated groups, and protein phosphatases (Erasers) facilitate the removal of the phosphoryl groups.^25^ Phosphorylation typically occurs on serine, threonine, or tyrosine residues within the protein structure.

### 2.2 Ubiquitination

Ubiquitination is a process wherein ubiquitin binds covalently to a target protein catalyzed by a series of enzymes. The C‐terminal glycine residue of ubiquitin forms a covalent bond with the amino group of the lysine side chain of other protein molecules. This process involves three enzymes (Writers) working together in a cascade: E1 ubiquitin‐activating enzyme, E2 ubiquitin‐conjugating enzyme, and E3 ubiquitin‐conjugating enzyme. Ubiquitination is a tightly regulated and reversible process. Deubiquitinating enzymes (Erasers) can reverse ubiquitination modifications by hydrolyzing peptide or isopeptide bonds between ubiquitin molecules or between ubiquitin and substrate proteins.^26^ In addition, proteins (Readers) that recognize ubiquitinated proteins by their ubiquitin‐binding domains are involved in the coordinated activity of ubiquitination.^27^

### 2.3 Acetylation

Acetylation modifications of proteins encompass both histone acetylation and nonhistone acetylation. Histone acetylation, pioneered by Vincent Allfrey in 1964, involves the transfer of the acetyl group from acetyl coenzyme A to specific lysine residues at the amino‐terminus of histones, facilitated by histone acetyltransferases (HATs). This process neutralizes the positive charge of lysine, leading to relaxation of the nucleosome structure and activation of transcription (lysine +1 to 0). Histone acetylation is mediated by HATs (Writers), with brominated chains acting as readers for read recognition. Conversely, deacetylation, catalyzed by histone deacetylases (HDACs, Erasers), is associated with transcriptional repression. Additionally, acetylation modifications extend beyond histones to include nonhistone proteins, playing diverse roles in various biological processes.^28^

### 2.4 Methylation

Methylation stands as a pivotal form of epigenetic regulation, involving the transfer of reactive methyl groups to target molecules catalyzed by methyltransferases (Writers). Histone methyltransferases are divided into two groups based on their structure and modification sites: histone lysine methyltransferases and protein arginine methyltransferases. Protein methylation predominantly occurs on arginine and lysine residues of histones and nonhistone proteins. Histone methylation primarily targets H3 and H4, where arginine residues can undergo monomethylation and demethylation, while lysine residues can be monomethylated, dimethylated, and trimethylated.^29^ Nonhistone methylation, on the other hand, typically modulates signal transduction and serves as a crucial regulator of cellular signaling.^30^

The counterpart to methylation is demethylation, facilitated by members of the Jumonji C‐terminal structural domain family and lysine‐specific demethylase 1 primarily responsible (Erasers) for lysine demethylation. Additionally, certain members of the Jumon C‐terminal structural domain family are known to catalyze arginine demethylation.^29^

### 2.5 SUMOylation

SUMO, the small ubiquitin‐like modifier, is a highly conserved molecule.^31^ It conjugates to lysine residues in proteins via isopeptide linkage, a process catalyzed by SUMO‐specific activating enzyme (E1), conjugating enzyme (E2), and ligase (E3) (Writers).^32^ Mammalian cells contain five SUMO isoforms: SUMO1, SUMO2, SUMO3, SUMO4, and SUMO5.^33^ SUMOylation regulates cellular transcription, nuclear integrity, proliferation, and senescence.^33^ DeSUMOylation is mainly mediated by SUMO‐specific peptidases (SENPs, Erasers).

### 2.6 Glycosylation

Protein glycosylation entails the covalent bonding of monosaccharides or glycans to particular residues of targeted proteins. Within eukaryotic cells, this process predominantly unfolds across the secretory pathway, initiating in the endoplasmic reticulum (ER) and culminating in the Golgi apparatus.^34^ Protein glycosylation encompasses both N‐terminal and O‐terminal glycosylation.

### 2.7 The lysine modifications

#### 2.7.1 Succinylation

Succinylation entails the covalent attachment of succinyl groups to lysine residues facilitated by succinyl donors mediated by succinyl‐coenzyme A.^35^ Succinylation accompanied by changes in amino acid charge (lysine +1 to −1). This modification can significantly impact enzymatic processes and metabolic pathways, particularly within mitochondrial metabolic pathways, thereby exerting diverse effects on cellular metabolism. Such effects extend to crucial pathways including the tricarboxylic acid cycle, electron transport chain, glycolysis, ketone body formation, fatty acid oxidation, and the urea cycle.^36^

#### 2.7.2 Crotonylation

Crotonylation, a lesser‐known protein modification, has gained attention in metabolic diseases. First identified in 2011,^37^ lysine crotonylation occurs not only in core histones but also in a variety of nonhistone proteins across a wide range of organisms. Like other PTMs, protein crotonylation is reversible and has been implicated in processes such as DNA damage and repair,^38^ tissue damage,^39^ inflammation,^40^ as well as self‐renewal and differentiation of stem cells.^41^ Among the various types of protein crotonylation, lysine crotonylation predominates, with HATs, like P300/CBP, PCAF, and MOF being the main enzymes responsible for catalyzing crotonylation reactions, while HDACs (HDAC1/2/3) and sirtuins (SIRT1/2/3) function to reverse these modifications.^42^

#### 2.7.3 Lactylation

Lactylation, the modification of lysine residues on histones by lactic acid during cellular metabolism, represents an important mechanism through which lactic acid exerts its functions. This modification plays a pivotal role in various cellular activities, including glycolysis‐related functions, macrophage polarization, vascular regulation, mitochondrial function, and nervous system regulation. The involvement of lactylation in these crucial processes suggests promising avenues for research in fields such as oncology and immunology.

#### 2.7.4 Malonylation

Protein malonylation, identified in 2011, predominantly occurs on lysine residues.^43^ The enzymatic machinery responsible for catalyzing protein malonylation includes malonyl coenzyme A, malonyl coenzyme A decarboxylase, and SIRT5.

### 2.8 The cysteine modifications

#### 2.8.1 Palmitoylation

Palmitic acid undergoes conversion to palmitoyl coenzyme A facilitated by palmitoyl acyl transferase, which is covalently bound to the sulfhydryl group of the C‐terminal cysteine residue of proteins through an unstable thioester bond, a process known as S‐palmitoylation. Notably, palmitoylation represents a reversible protein modification, with the reverse process catalyzed by a family of serine hydrolases B.^44^

#### 2.8.2 S‐sulfhydration

S‐sulfhydration, alternatively known as persulfation, represents a novel PTM mechanism involving the modification of cysteine residues through the utilization of hydrogen sulfide (H2S) or persulfide.^45^

## 3 PROTEIN PTMs IN DM

**FIGURE 1:**
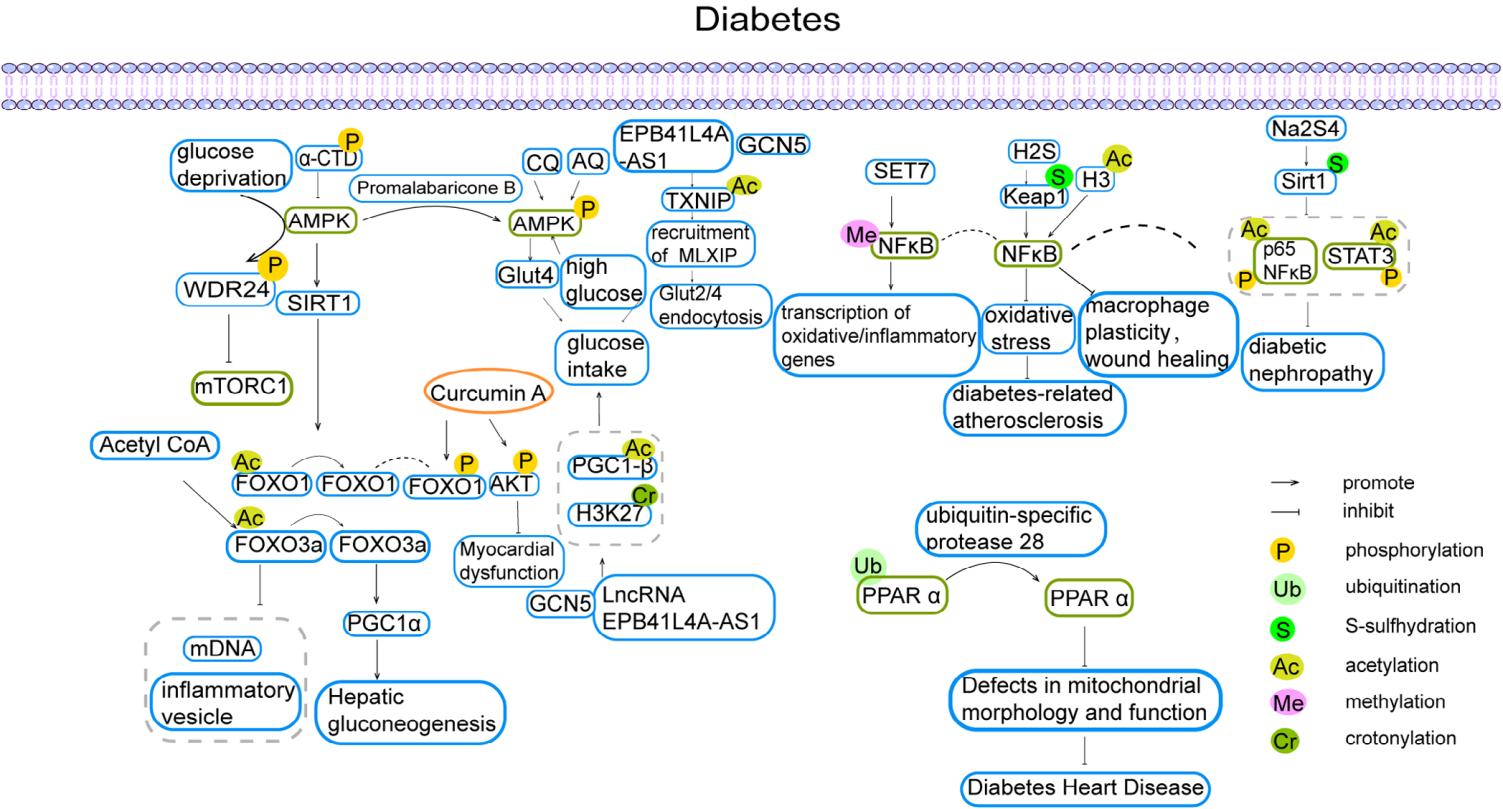
PTMs in DM. Posttranslational modifications (PTMs) of proteins are closely related to the development of diabetes. (A) Deubiquitination of peroxisome proliferator‐activated receptor (PPAR) inhibits diabetic heart disease. (B) Methylation of nuclear factor kappa‐B (NF‐κB) promotes transcription of inflammation‐related genes. Acetylation and phosphorylation of NF‐κB are associated with diabetic neuropathy. Acetylated H3 and sulfated Keap1 modulate the activity of NF‐κB, thereby affecting oxidative stress. (C) AMP‐activated protein kinase (AMPK) promotes hepatic gluconeogenesis by promoting the phosphorylation of WD repeat domain 24 (WDR24), inhibits mammalian target of rapamycin complex 1 (mTORC1). AMPK promotes hepatic gluconeogenesis by promoting deacetylation of Forkhead box O (FOXO). The phosphorylation of AMPK itself is regulated by rosemary extract, chloroquine (CQ), amodiaquine (AQ), hyperglycemia, and affects glucose uptake.

The prevalence of DM is increasing year by year and is accompanied by numerous complications that are increasingly threatening human public health. Current evidence suggests that PTMs of proteins play an important role in diabetes and its complications (Figure 1).^46^

### 3.1 Phosphorylation in DM

Phosphorylation plays a crucial role in the pathogenesis of DM. Phosphorylation mediates this disease by affecting the insulin signaling pathway, AMP‐activated protein kinase (AMPK) signaling pathway, and others. The dynamic interplay of phosphorylation and dephosphorylation processes involving the insulin receptor and its downstream proteins significantly impacts energy metabolism. Glucose present in the bloodstream stimulates insulin secretion from pancreatic β‐cells, which then travels through the bloodstream to tissues, activating the insulin receptor. This activation initiates a cascade of phosphorylation‐dephosphorylation processes.^47^, ^48^ The insulin receptor consists of α and β subunits, with the β subunit containing a structural domain expressing insulin‐stimulated kinase activity. Mutations at phosphorylation sites (sites 1158, 1163, and 1162) of the β subunit impair kinase activity, thereby reducing the metabolic effects of insulin. In muscle and liver, insulin receptor substrate‐1 (IRS‐1) and IRS‐2 act as major docking proteins, respectively, undergoing tyrosine phosphorylation in response to insulin receptor tyrosine kinase.^49^ Phosphorylation of IRS‐1 is inhibited by saturated fatty acid‐mediated ER stress, further impacting insulin signaling and sensitivity.^50^, ^51^ Additionally, diacylglycerol activates protein kinase Cθ in muscle and protein kinase Cε in the liver, promoting phosphorylation of IRS‐1 at Ser1101 and insulin receptor at Thr1160, consequently inhibiting insulin signaling.^50^ Inhibition of vascular endothelial protein tyrosine phosphatase dephosphorylates endothelial nitric oxide synthase at Tyr81, thereby enhancing diabetes‐induced endothelial function and lowering blood pressure.^52^ Moreover, glycogen synthase kinase 3 beta (GSK3β) regulates the phosphorylation of mitsugumin 53 at S255, a process induced by aberrant insulin signaling in diabetic mice. Consequently, inhibition of the insulin receptor–IRS1–GSK3β–mitsugumin 53 pathway protects diabetic hearts in mice.^53^

AMPK serves as a critical cellular energy sensor, playing a pivotal role in regulating glucose metabolism and uptake in DM. Comprising three subunits, α, β, and γ, phosphorylation of serine 485/491 on the C‐terminal structural domain of the α subunit can modulate AMPK activity.^54^ Phosphorylation of AMPK enhances glucose uptake in muscle cells and reduces blood glucose levels by upregulating glucose transporter protein 4 (GLUT4) expression.^55^, ^56^ Chloroquine and amodiaquine have been shown to alleviate diabetic tubulopathy by promoting AMPK phosphorylation in renal tubular cells of diabetic nephropathic mice.^57^ High glucose levels induce reactive oxygen species production, leading to AMPKα phosphorylation at S485/491 and the recruitment of E3 ligase mitsugumin 53.^58^ Fisetinone inhibits mitochondrial superoxide production and reduces AMPK phosphorylation in the kidneys of diabetic mice.^59^ Moreover, glucose deprivation inhibits mechanistic target of rapamycin complex 1 (mTORC1) activation by promoting AMPK‐mediated phosphorylation of WD repeat domain 24 at S155.^60^

### 3.2 Ubiquitination in DM

Ubiquitination plays a role in the development and progression of DM mainly by affecting the IRS signaling pathway and the ubiquitin–proteasome system. First, ubiquitin deficiency leads to the downregulation of IRS signaling, which is associated with the production of suppressor of cytokine signaling 1 and suppressor of cytokine signaling 3 induced by inflammatory cytokines. Second, it results in the downregulation of insulin receptor. Additionally, ubiquitination is associated with impaired insulin biological responses.^61^

The ubiquitin–proteasome system serves as the principal pathway for cellular protein degradation, with the multicatalytic complex 26S proteasome recognizing and degrading ubiquitin‐tagged proteins. Studies have revealed elevated levels of ubiquitination in proteins within β‐cells of patients diagnosed with T2DM.^62^ Additionally, reductions in ubiquitin–proteasome system components have been observed in retinal cells of diabetic rats, with a concomitant decrease in ubiquitin‐activating enzyme E1 during induced ER stress.^63^ S‐phase kinase‐associated protein 2 ubiquitin ligase, a receptor component of the SCF ubiquitin ligase complex, facilitates ubiquitination and degradation of p27, a protein closely linked to β‐cell production and insulin resistance.^64^ Defects in protein degradation within β‐cells of T2DM patients have been associated with misfolding of pancreatic amyloid‐derived polypeptide h‐IAPP, leading to diminished levels of the deubiquitinating enzyme UCH‐L1 and consequent impairment of β‐cell function.^65^ Furthermore, the ubiquitin‐specific protease has been shown to mitigate diabetic heart disease in mice by deubiquitinating and stabilizing peroxisome proliferator‐activated receptor α (PPARα), thereby inhibiting mitochondrial morphofunctional defects.^66^ In a randomized controlled trial, it was found that the PPARγ activator rosiglitazone suppressed ubiquitin–proteasome activity in atherosclerotic lesions in diabetic patients, potentially through the downregulation of NF‐κB‐mediated inflammatory pathways.^67^

### 3.3 Acetylation in DM

In the context of DM, HDACs and HATs influence maturity‐onset diabetes of the young (MODY) by modulating the acetylation levels of relevant factors. Deacetylation of Forkhead box O3a (FOXO3a), liver X receptor alpha (LXRα), and signal transducer and activator of transcription 3 (STAT3) mediated by the deacetylase Sirtuin family is implicated in DM. Furthermore, protein acetylation regulates glucose uptake.

The activity of HDACs or HATs plays a pivotal role in the transcriptional regulation of genes, thereby influencing cellular function and metabolic homeostasis.^68^ MODY is a monogenic chromosomal form of T2DM. Acetylation of hepatocyte nuclear factor 4α (HNF4α) associated with MODY1 is regulated by CBP.^69^ The expression of glucokinase (GCK) associated with MODY2 is regulated by HNF4α and involves transcriptional complexes containing HAT activity. HNF1α associated with MODY3 affects the hyperacetylation of nucleosomes on the promoters of the GLUT2 and pyruvate kinase L/R genes in pancreatic islets.^70^ MODY4 development is linked to mutations in pancreatic and duodenal homology cassette‐1/insulin promoter factor‐1, which indirectly regulates insulin production and is regulated by the acetylation of histones H3 and H4.^71^ MODY5 development is associated with mutations in the HNF 1/transcription factor 2 gene, the activity of which may be influenced by HDAC1.^72^ MODY6 is associated with neuronal differentiation 1, and HAT regulates insulin transcription through direct acetylation of neuronal differentiation 1.^73^

Palmitate induces acetylation of FOXO3a, leading to downregulation of ATP‐induced mitochondrial DNA release and inflammatory vesicle activation in diabetic macrophages.^74^ SIRT6 inhibits notch receptor 3 and notch receptor 9 transcription by deacetylating histone, thereby preventing podocyte injury and diabetic nephropathy.^75^ Animal experiments have demonstrated that SIRT3 reduces diabetic neuropathic pain by deacetylating and stabilizing FOXO3a, inhibiting oxidative stress.^76^ Diabetic Goto‐Kakizaki rats exhibit significant cardiac hypertrophy and dysfunction, associated with the activation of FOXO3a–SIRT1.^77^ SIRT1 plays a crucial role in glucose metabolism. Fasting promotes SIRT1‐mediated deacetylation of LXRα to alleviate diabetic retinopathy.^78^ Fasting induces gluconeogenic genes and hepatic glucose output by promoting hepatic SIRT1‐mediated deacetylation of peroxisome proliferative‐activated receptor, gamma, coactivator 1 alpha (PGC‐1α).^79^ SIRT1 mediates the deacetylation of STAT3, and baicalein can maintain the acetylation level of STAT3 by downregulating SIRT1 activity and inhibiting downstream PGC‐1α, thus inhibiting hepatic gluconeogenesis.^80^ AMPK controls energy metabolism in mouse skeletal muscle by activating SIRT1, leading to deacetylation of FOXO1 and FOXO3a.^81^

Glucose uptake is intricately regulated by protein acetylation mechanisms. For instance, the binding of IncRNA EPB41L4A‐AS1 to HAT GCN5 enhances acetylation at histone marks histone H3 lysine 27 (H3K27) and H3K14 within the promoter region of thioredoxin interacting protein, facilitating transcriptional activation. This process is mediated by the recruitment of the transcriptional activator MLX interacting protein, which enhances the endocytosis of GLUT2/4 transporters, ultimately leading to the inhibition of glucose uptake. Conversely, EPB41L4A‐AS4 modulates the nonhistone acetylation of PGC1β in the GLUT3 promoter region through its interaction with GCN1. This interaction results in the suppression of GLUT4 transcription and subsequent glucose uptake by muscle cells.^82^ Moreover, diabetic patients with increased protein glycation exhibit reduced aspirin‐mediated protein acetylation.^83^

The cyclin‐dependent kinase 4 (CDK4) nucleotide substitution confers protection to CDK4 from inhibition by the INK family of cell cycle inhibitors, thereby ameliorating glucose intolerance in IRS2‐deficient mice. Conversely, CDK4 inhibits the expression of pancreatic and duodenal homeobox 1 via the insulin signaling mediator FOXO1. This inhibition is associated with deacetylation and reduced abundance of FOXO1.^84^ Additionally, lysine acetyltransferase KAT8, also known as MOF, mediates the acetylation of histone H4 at lysine 16 (H4K16ac). Dysfunction of MOF in mice leads to an imbalance in glucolipid metabolism and an increased risk of developing T2DM.^85^

### 3.4 Methylation in DM

Protein methylation levels play a crucial role in the development of diabetes and its associated complications. Diabetic vascular complications (DVC) encompass both macrovascular and microvascular diseases, including cardiovascular disease, diabetic nephropathy, retinopathy, and neuropathy.^86^ DVC significantly diminishes quality of life, imposes substantial economic burdens on individuals and society, and stands as the leading cause of death in individuals with diabetes.^87^ Gene regulation mediated by histone methylation on arginine and lysine residues is implicated in the pathogenesis of DVC. Mice exhibiting diabetic nephropathy demonstrated a consistent decrease in histone H3 lysine 27 trimethylation (H3K27m3), a silencing mark, and a concurrent increase in the level of H3 lysine 4 dimethylation, an activation mark. Moreover, there was an elevation in the abundance of H3K27m3 and the demethylase KDM6A in the kidney.^88^

Histone methyltransferase SET7 is upregulated in peripheral blood mononuclear cells from patients with T2DM, where it mediates the monomethylation of H3K4 at the nuclear factor kappa‐B (NF‐κB) p65 promoter. This epigenetic alteration is associated with the upregulation of NF‐κB, transcription of oxidative/inflammatory genes, and elevated plasma levels of intercellular adhesion molecule‐1 and monocyte chemotactic protein‐1.^89^ In diabetic patients, SET domain bifurcated histone lysine methyltransferase 2 is impaired, resulting in decreased trimethylation levels of histone 3 and increased NF‐κB‐mediated inflammation. SET domain bifurcated histone lysine methyltransferase 2 deficiency also impacts macrophage plasticity and wound healing.^90^ Additionally, hepatic levels of H3K4 monomethylation and H3K9 dimethylation were found to be elevated in diabetic mice, which were mitigated by treatment with exonuclease‐4.^91^

### 3.5 SUMOylation in DM

Protein SUMOylation influences glucose tolerance and is associated with diabetic heart disease and diabetic neuropathy. Glyceraldehyde‐3‐phosphate dehydrogenase (GAPDH) maintains metabolic homeostasis through glycolysis and undergoes increased activity through SUMOylation. The key metabolic enzyme is SUMOylated, and activated activity of GAPDH at lysine 332.^92^ The activity of GAPDH is reduced by treatment with SUMO specific peptidase 1, a SUMO‐specific isopeptidase. Mice lacking SUMOylation exhibit exacerbated neuropathology and sensory loss in diabetic neuropathy.^92^

X‐box binding protein 1 (XBP1) is a crucial transcription factor for maintaining ER homeostasis, activated in response to disruptions in ER protein folding. Diabetic mice develop significant myocardial structural abnormalities, cardiac hypertrophy, and impaired cardiac function. Impaired nuclear translocation of XBP1 is observed in the hearts of diabetic mice. However, phosphorylation of XBP1 at S348 and SUMOylation of XBP1 at K276 promote its nuclear translocation, ameliorating the progression of diabetic cardiomyopathy.^93^ UBC9, as a SUMOylated E2‐coupled enzyme, knockout downregulates SUMOylation levels in pancreatic β‐cells, impairing glucose tolerance and leading to progressive destruction of β‐cells, ultimately resulting in fasting hyperglycemia and diabetes. Conversely, UBC9 overexpression upregulates sumoylation levels, preventing β‐cell loss while inhibiting β‐cell function, leading to glucose intolerance.^94^

### 3.6 Glycosylation in DM

Protein glycosylation plays a critical role in regulating insulin secretion and is intricately linked to diabetes and its various complications.

Abnormal N‐glycosylation of proteins, such as GLUT2 in pancreatic β‐cells, contributes to impaired insulin secretion and the development of T2DM. Furthermore, defects in N‐glycosylation of T‐cells are strongly correlated with the onset of T1DM.^95^

The involvement of protein glycosylation extends to various diabetic complications. Proteins undergo nonenzymatic modification by glucose, resulting in the formation of advanced glycosylation end products (AGEs), which are implicated in tissue damage. AGE proteins bind to their high‐affinity receptors, triggering degradation and clearance processes, along with the synthesis and secretion of potent cytokines. Accumulation of tissue AGEs contributes significantly to diabetic vasculopathy, highlighting the potential of AGE inhibitors like aminoguanidine hydrochloride as a therapeutic target for DM.^96^ Plasma protein N‐glycosylation patterns are associated with complications of T2DM. Recent meta‐analyses have revealed that elevated levels of certain glycans, such as bifurcation on dibasic glycans and increased 2,6‐glycosylation on ternary glycans, are strongly linked to the prevalence of CVD and diabetic nephropathy, respectively.^97^ Moreover, protein glycosylation is intricately involved in the pharmacodynamic mechanisms underlying the treatment of diabetes. Medications such as metformin and statins have been shown to modulate protein glycosylation, leading to decreased fucosylation and increased galactosylation and sialylation.^98^

### 3.7 The lysine modifications in DM

#### 3.7.1 Succinylation in DM

Succinylation plays a pivotal role in the pathogenesis of DM. In T2DM, pancreatic β‐cells, retinal cells, and kidneys are subjected to glucotoxicity, leading to elevated levels of succinylation. This elevation exacerbates oxidative stress, resulting in the inactivation of key enzymes and chaperones within cells, thereby promoting apoptosis and contributing to diabetic tissue damage.^99^

#### 3.7.2 Crotonylation in DM

Glucose uptake and insulin function are tightly regulated by protein crotonylation. EPB41L4A‐AS1 modulates histone H27K4 crotonylation in the GLUT3 promoter region, thereby epigenetically controlling the transcription of GLUT14 and thioredoxin interacting protein. Overexpression of EPB41L4A‐AS1 leads to a reduction in glucose uptake, while its knockdown enhances glucose uptake levels.^82^ Additionally, GFP‐W, a novel heteropolysaccharide derived from Ashwagandha, has shown potential as a hypoglycemic agent, with its antidiabetic activity associated with alterations in protein lysine crotonylation levels. GFP‐W enhances insulin sensitivity through regulation of protein lysine crotonylation.^100^ Diabetic kidney disease, a significant complication of diabetes, is a major contributor to chronic kidney disease.^101^ Histone crotonylation contributes to increased renal SIRT3 and PGC‐1α expression, while decreasing C‐C motif chemokine ligand 2 expression to safeguard the kidney from acute injury.^39^

#### 3.7.3 Malonylation in DM

In the context of DM, dysregulated levels of protein malonylation have been observed. Liver‐specific lysine malonylation emerges as the predominant acylation pattern in animal models of T2DM, such as db/db and ob/ob mice. Proteomic analysis reveals an enrichment of malonylated proteins in metabolic pathways, particularly those governing glucose and fatty acid metabolism.^102^ Protein malonylation appears to modulate enzyme activity in glucose metabolism, thus implicating its involvement in the pathogenesis of T2DM.^103^ Investigation into histone PTM sites in a pre‐diabetic mouse model unveils seven loci of malonylated proteins. However, the regulatory mechanisms governing gene expression at these sites remain elusive and warrant further investigation.^104^

### 3.8 The cysteine modifications in DM

#### 3.8.1 Palmitoylation in DM

In the context of DM, palmitoylation levels of key proteins such as GLUT4 and mucin 2 are significantly implicated. Impaired palmitoylation of GLUT4 adversely affects insulin‐stimulated glucose uptake in muscle and adipose tissue, representing a critical feature of insulin resistance.^105^ Among the palmitoyltransferases, DHHC7 emerges as a pivotal mediator of GLUT4 palmitoylation. DHHC7 deficiency hampers insulin‐dependent GLUT4 membrane translocation by modulating the palmitoylation status of GLUT4.^106^ Moreover, fatty acid synthase (FAS), an insulin‐responsive enzyme crucial for de novo lipogenesis, undergoes induction by insulin. However, in diabetic states, FAS levels are diminished while intestinal permeability is heightened. Functionally, FAS aids in maintaining the intestinal mucus barrier integrity by facilitating S‐palmitoylation of mucin 2 at its N‐terminus.^107^

#### 3.8.2 S‐sulfhydrationin in DM

S‐sulfhydration of SIRT1 and Kelch‐like ECH‐associated protein 1 (KEAP1) plays a significant role in diabetic complications, particularly in the context of diabetic nephropathy, a critical complication of DM. SIRT1, when sulfhydrated by Na2S4, exerts a protective effect against diabetic nephropathy by impeding the phosphorylation and acetylation of p65 and STAT3.^108^ Similarly, H2S sulfhydrates KEAP1 at Cys151, leading to the activation of nuclear factor erythroid 2‐related factor 2 (NRF2) signaling. This activation helps to alleviate oxidative stress and mitigate diabetes‐related atherosclerosis.^109^

### 3.9 Drugs targeting PTM in DM

#### 3.9.1 SGLT‐2 inhibitor

Phosphorylation of Kelch like family member 3 at serine 433 was detected in the kidneys of db/db mice. Treatment with a sodium‐glucose cotransporter‐2 (SGLT‐2) inhibitor reduced protein kinase C activity and decreased the phosphorylation level of Kelch like family member 3 at serine 433, thereby improving T2DM.^110^, ^111^ Additionally, the SGLT‐2 inhibitor empagliflozin was found to enhance inflammation resolution and alleviate diabetic microvascular injury by modulating AMPK‐mediated phosphorylation of dynamin related protein 1.^112^, ^113^

#### 3.9.2 GLP‐1 agonist

The glucagon‐like peptide‐1 receptor (GLP1R) agonist exenatide effectively regulates blood glucose levels in patients with T2DM by enhancing glucose‐dependent insulin secretion.^114^ Animal experiments have demonstrated that exendin‐4 promotes the phosphorylation of hepatic AMPK and acetyl‐CoA carboxylase in diet‐induced obesity mice.^115^ Clinical studies have corroborated the efficacy of GLP1R agonist treatment in reducing appetite and body weight in patients with T2DM.^115^, ^116^ Furthermore, a randomized placebo‐controlled crossover study revealed that liraglutide administration increased AMPK phosphorylation and mitigated peripheral insulin resistance.^117^ A meta‐analysis showed that GLP1R agonist treatment significantly reduced renal composite outcomes by reducing urinary albumin excretion.^118^

#### 3.9.3 GCK agonist

GCK plays a pivotal role in β‐cell glycolysis, facilitating the phosphorylation of glucose to glucose 6‐phosphate. It serves as a critical rate‐limiting enzyme, regulating insulin secretion. GCK agonists exert their effects by promoting insulin secretion and enhancing hepatic glucose uptake.

Dorzagliatin, a dual‐acting GCK activator, represents a significant milestone as the first NDA filing for the treatment of DM. A phase 2 clinical study of Dorzagliatin as a monotherapy for Chinese patients with T2DM showcased beneficial outcomes in terms of glycemic control, safety, and tolerability.^119^ Subsequent phase 3 clinical trials further underscored the favorable efficacy and safety profile of Dorzagliatin,^120^ either as an initial treatment for T2DM or as an add‐on therapy to metformin.^121^

#### 3.9.4 SIRT1 activator

The SIRT1 activator SRT3025 has demonstrated efficacy in attenuating cell proliferation, reducing plasma glucagon concentration, and improving glycemic control in diabetic mice.^122^ Additionally, SRT3025 has been shown to enhance glycemic control, beta‐cell mass, and plasma insulin concentration in obese type 2 diabetic mice.^123^ In a double‐blind randomized controlled trial involving 71 patients with T2DM, resveratrol was found to significantly increase the levels of p53 and p21 while decreasing the serum cluster of differentiation 163 to TNF‐like weak apoptosis inducer ratio.^124^ Moreover, resveratrol has been shown to promote the phosphorylation level of AMPK, thereby enhancing its activity.^125^ Furthermore, SRT1720 has been demonstrated to improve renal function and mitigate renal pathologies including glomerular hypertrophy, thylakoid dilatation, glomerulosclerosis, and interstitial fibrosis in diabetic mice through modulation of the SIRT1/HIF1α/GLUT1 pathway.^126^ Additionally, SRT1720 ameliorates diet‐induced insulin resistance, possibly through direct deacetylation of PGC‐1α, FOXO1, and p53.^127^

#### 3.9.5 HDAC inhibitor

The HDAC inhibitor NaB has been shown to promote pluripotent human embryonic stem cells to generate insulin‐producing islet‐like cell clusters.^128^ Furthermore, NaB exhibits the ability to attenuate the hyperglycemia‐induced inflammatory response by downregulating the acetylation level of NF‐κB p65 in human monocytes.^129^ Valproic acid has been identified to enhance pancreatic endoderm formation and induce the differentiation of adipose‐derived stem cells into insulin‐secreting cells. HC toxin, another HDAC inhibitor, has demonstrated efficacy in enhancing β‐cell function in primary mouse and human pancreatic islets. This effect is mediated by regulating the accumulation of IRS‐1 and promoting AKT serine/threonine kinase (AKT) phosphorylation.^130^

#### 3.9.6 Others

O‐GlcNAc modification on AKT affects AKT phosphorylation, leading to impaired activation and thus glucose metabolism.^131^ Animal experiments showed that the transferase inhibitor ST045849O‐GlcNAc reduced gluconeogenesis.^132^

## 4 PROTEIN PTMs IN OBESITY

**FIGURE 2:**
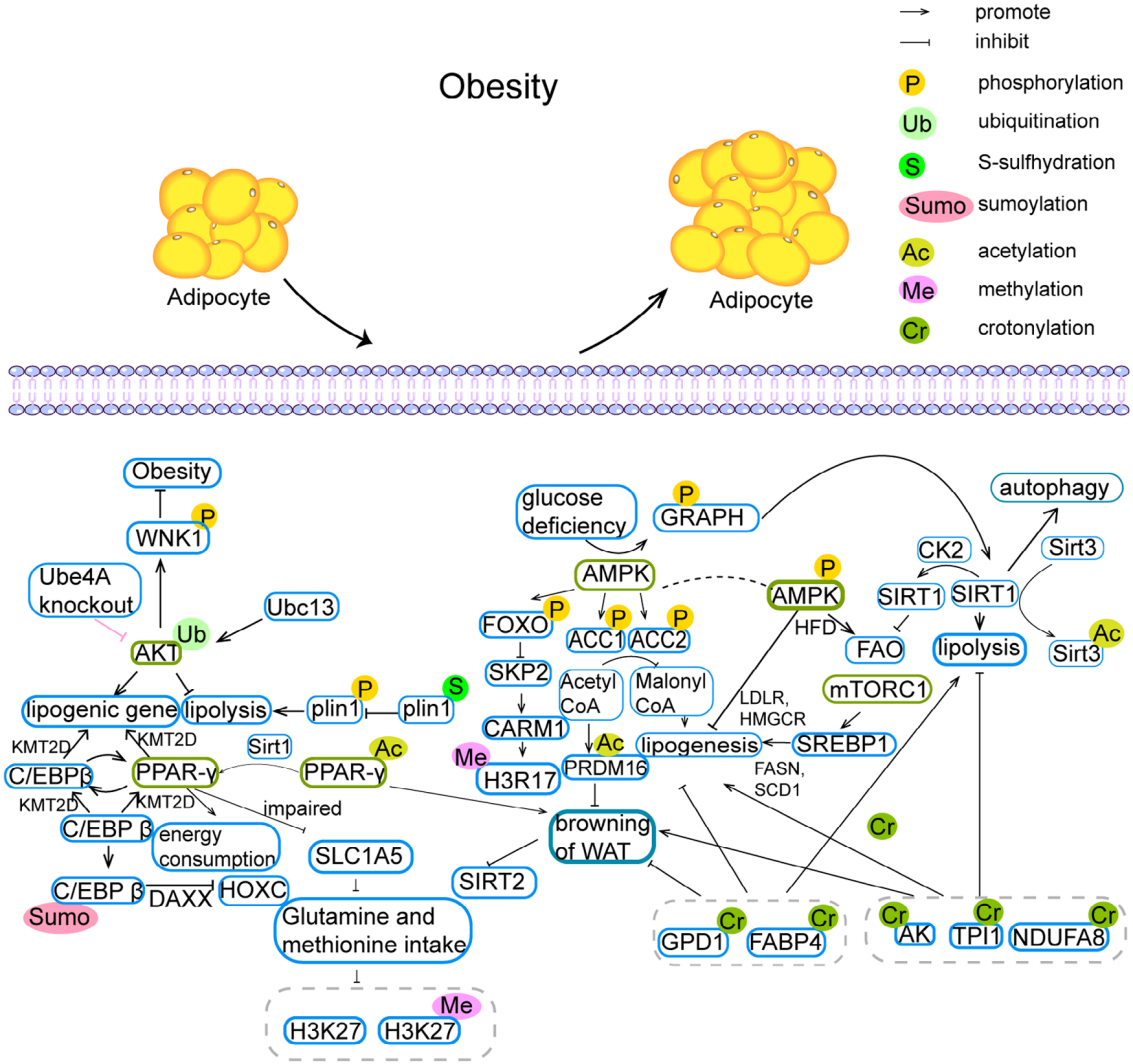
PTMs in obesity. Multiple protein posttranslational modifications mediated obesity. A variety of protein posttranslational modifications mediate the development of obesity. (A) Ubiquitination of protein kinase B (AKT) and deacetylation of peroxisome proliferator‐activated receptor γ (PPARγ) are associated with transcription of lipogenic genes and lipolysis. (B) AMP‐activated protein kinase (AMPK) is another important signaling pathway in the process of obesity development that is regulated by posttranslational modifications (PTMs). On the one hand, AMPK promotes the phosphorylation of acetyl‐CoA carboxylase (ACC), which is correlated with the browning of white adipose tissue (WAT). On the other hand, the level of phosphorylation of AMPK itself is associated with fatty acid oxidation and lipogenesis.

Obesity is a major public health challenge facing the world today. It is not only a stand‐alone health problem, but also a risk factor for a variety of chronic diseases, and it is particularly important to understand the pathogenesis of the disease and effectively combat it. PTMs of protein are important parts of obesity pathogenesis (Figure 2).

### 4.1 Phosphorylation in obesity

Phosphorylation modifications exert a significant impact on obesity, primarily Phosphorylation affects lipogenesis and metabolism mainly by influencing sterol regulatory element‐binding protein (SREBP), the sirtuin family of deacetylases, and AMPK pathways.

SREBP1 orchestrates the expression of lipogenic genes, contributing to the development of obesity. CREB‐regulated transcriptional coactivator 2 interacts with the COPII complex, competitively disrupting COPII‐dependent SREBP1 transport, thereby modulating lipid metabolism.^133^ Mammalian target of rapamycin (mTOR), a serine/threonine protein kinase of the PIKK family, regulates the phosphorylation of CREB‐regulated transcriptional coactivator 2, subsequently downregulating the expression of lipogenic genes.^134^ Phosphorylation of mTOR further contributes to lipid metabolism disorders by modulating SREBP1.^135^ Activated AKT in hepatocellular carcinoma cells promotes the phosphorylation of cytosolic phosphoenolpyruvate carboxykinase 1, which translocates to ER and phosphorylates insulin induced gene 1 and insulin induced gene 2. This cascade of phosphorylation events activates SREBP1 or SREBP2, leading to the transcription of downstream adipogenesis‐related genes.

SIRT1, a NAD‐dependent deacetylase, undergoes phosphorylation at Ser‐164 catalyzed by casein kinase 2, inhibiting its nuclear localization in obese mice.^136^ Decreased SIRT1 activity impairs fatty acid oxidation and exacerbates hepatic steatosis.^136^ Conversely, phosphorylation of SIRT1 at Ser‐46 by c‐Jun N‐terminal kinase 1 activates SIRT1 function, ameliorating hepatic steatosis in obesity.^137^ Glucose deficiency promotes AMPK‐induced phosphorylation of GAPDH, activating SIRT1 and initiating autophagy.^138^ SIRT1 overexpression in the striatum and hippocampus promotes fat accumulation and upregulates lipogenic gene expression in white adipose tissue (WAT).^139^ Additionally, nuclear factor 1 A improves glucose homeostasis, enhances energy expenditure for adaptive thermogenesis, and mitigates obesity by promoting mitochondrial oxidative phosphorylation and inhibiting inflammation.^140^

Treatment with canagliflozin for 8 weeks has been shown to promote AMPK phosphorylation and induce mitochondrial remodeling in WAT in mice.^141^ Specifically, phosphorylation of AMPKβ1 at Ser108 has been linked to the promotion of fatty acid oxidation and mitochondrial biogenesis.^142^ Furthermore, tschimganidine has been found to increase AMPK phosphorylation, thereby reducing adipogenesis.^143^

### 4.2 Ubiquitination in obesity

The functions of ubiquitination and ubiquitination‐related enzymes within the AKT signaling pathway exert significant influence on lipid metabolism. Ubiquitination of K63 stands out as a pivotal aspect of AKT activation in insulin signaling. G protein pathway suppressor 2 plays a crucial role in preventing AKT ubiquitination by inhibiting the activity of the ubiquitin‐conjugating enzyme Ubc13. Consequently, deficiency of G protein pathway suppressor 2 in adipose tissue in mice leads to constitutive ubiquitination and subsequent AKT activation, resulting in disrupted lipid metabolism, increased obesity, and elevated levels of lipocalin.^144^ Upon induction by insulin, AKT undergoes ubiquitination and activation in the presence of adaptor protein phosphotyrosine interacting with PH domain and leucine zipper 1. Knockdown of ubiquitination factor E4A inhibits this ubiquitination process in mice.^145^ Enhanced expression of the ubiquitin editing enzyme A20 has been shown to suppress adipogenesis and the expression of key markers associated with adipogenesis, concomitant with attenuated activation of p38 and AKT signaling.^146^ AKT3 facilitates lysine‐free protein kinase‐1 specific phosphorylation and its subsequent degradation via the ubiquitin–proteasome pathway, thereby alleviating high‐fat diet (HFD)‐induced obesity in mice.^147^

Deletion of the ubiquitin E3 ligase CUL2–APPBP2 enhances protein stability of PR/SET domain 2, counteracting diet‐induced obesity and ameliorating insulin resistance and dyslipidemia.^148^ Furthermore, the deubiquitinase ubiquitin‐specific protease 1 promotes lipid accumulation by deubiquitinating and upregulating the level of CCAAT/enhancer‐binding protein β, thus inducing adipogenesis.^149^

### 4.3 Acetylation in obesity

Lysine deacetylases, such as HDACs, play pivotal roles in fat differentiation and production. The acetylation levels of PPAR also exert profound effects on lipid metabolism and homeostasis. Various isoforms of HDACs exhibit distinct effects on lipid metabolism. HDAC1 or HDAC2 positively regulate lipid production, as evidenced by reduced lipid levels in mice deficient in these HDAC isoforms. Conversely, SIRT1, SIRT2, and HDAC9 negatively regulate lipid content. SIRT1 promotes lipid mobilization, with its overexpression leading to reduced adipogenesis. In contrast, alterations in SIRT2 expression either inhibit or promote adipogenesis. Knockdown of HDAC9 is a crucial factor in adipocyte differentiation and adipogenesis, accelerating the process in mice.^150^

SIRT1 enhances thermogenesis and energy expenditure by promoting WAT expression akin to brown adipose tissue (BAT) through deacetylation of PPARγ.^151^ Elevated levels of histone acetylation have been observed in obese children, potentially linked to the downregulation of SIRT1 levels in vivo.^152^ Mice with SIRT1 adipocyte‐specific knockout display increased adipogenesis.^153^ Additionally, SIRT1 mediates the deacetylation of SIRT3 to mitigate obesity.^154^

Obesity is strongly correlated with disrupted circadian rhythms. Obese mice exhibit altered circadian rhythms of PPARγ acetylation, thereby impacting metabolic rhythms.^155^ Mechanistically, impaired PPARγ downregulates solute carrier family 1 member 5 level, leading to reduced glutamine and methionine uptake by adipocytes, as well as decreased levels of H3K27ac and H3K4me3 on the promoter of basic helix‐loop‐helix ARNT like 1.^156^ Mice harboring a PPARγ acetylation mutation display a white phenotype in BAT.^157^ Acetylation of PR structural domain‐containing protein 16 by acetyl‐CoA derived from branched keto acids inhibits WAT browning. Disrupting the interaction between PR structural domain‐containing protein 16 and PPARγ helps maintain WAT properties.^158^

### 4.4 Methylation in obesity

Protein methylation levels play a crucial role in the development of obesity. Findings from a Mendelian randomization study involving 1021 participants suggest a causal association between the methylation of Janus kinase 2 (JAK2) and obesity.^159^ Histone‐lysine N‐methyltransferase 2D is indispensable for adipogenesis, acting as the primary H3K4 single and double methyltransferase on enhancers related to lipogenesis and myogenesis. Histone‐lysine N‐methyltransferase 2D facilitates the activation of cell‐type‐specific enhancers during both lipogenesis and myogenesis processes.^160^ Nicotinamide N‐methyltransferase plays a significant role in histone methylation, polyamine fluxes, and the regulation of SIRT1 through the modulation of NAD and S‐adenosylmethionine levels in adipose tissue. Knockdown of nicotinamide N‐methyltransferase effectively prevents diet‐induced obesity.^161^ Additionally, nutrient restriction triggers AMPK‐dependent phosphorylation of FOXO3a, resulting in increased dimethylation of histone H3 Arg17 due to transcriptional inhibition of S‐phase kinase‐associated protein 2, which upregulates the level of arginine methyltransferase 1.^162^

### 4.5 SUMOylation in obesity

Protein SUMOylation plays a crucial role in regulating lipid production and storage. UBC9, a key enzyme in the SUMOylation pathway, exhibits significant upregulation in visceral adipocytes of both obese patients and mice. Mice with UBC9 knockout display a remarkable reduction in weight gain, and adipocyte‐specific UBC9 deficiency protects against high‐fat diet‐induced obesity, insulin resistance, hepatic steatosis, and inflammation. Mechanistically, UBC9 mediates the SUMOylation of ER protein 44 at K76, thereby enhancing its stability and protecting it from ubiquitin‐mediated degradation. This process exacerbates adipocyte ER stress and insulin resistance.^163^

Adipocyte‐specific knockout of SENP2 confers resistance to diet‐induced obesity. Inhibition of homeobox C10 is crucial for adipocyte differentiation in the inguinal WAT of SENP2‐knockout mice. CCAAT/enhancer‐binding protein β regulates homeobox C10 expression in a SUMOylation‐dependent manner.^164^ Furthermore, SENP2 regulates adipose lipid storage by de‐SUMOylating SET domain bifurcated histone lysine methyltransferase 1.^165^

### 4.6 Glycosylation in obesity

AGE‐specific receptor (AGER) activity plays a pivotal role in the development of obesity. Autophagy is intricately linked with normal intracellular lipid metabolism, where HMGB1 enhances autophagy by binding to the AGER, thus mitigating cell death.^166^ In HFD‐induced obese mice, adipocytes display elevated expression of AGER. Conversely, adipose tissue in AGER‐deficient mice demonstrates a mitigated phenotype, characterized by reduced adipocyte hypertrophy, diminished macrophage infiltration, and improved insulin sensitivity.^167^ Notably, oligofructose, utilized in the management of diet‐induced obesity, elicits an increase in the expression of genes associated with mucus production, glycosylation, and secretion^168^

### 4.7 The lysine modifications in obesity

#### 4.7.1 Succinylation in obesity

Succinylation levels of proteins in BAT influence lipid metabolism. BAT is crucial for regulating energy expenditure, thermogenesis, and glucose homeostasis, with its abundance negatively correlated with body mass index, suggesting a potential role in metabolism^169^ SIRT5 modulates succinylation and malonylation of mitochondrial proteins in BAT, and its knockout in BAT leads to metabolic inflexibility and disrupts mitochondrial homeostasis^170^ Recent studies have revealed that allicin activates BAT and enhances energy expenditure. Allicin significantly increases the succinylation level of uncoupling protein 1 (UCP1) in BAT by inhibiting SIRT5.^171^

#### 4.7.2 Crotonylation in obesity

Crotonylation also plays a pivotal role in obesity, particularly in the browning of WAT and its impact on fat thermogenesis. WAT, responsible for energy storage, can be induced to undergo browning, a process that enhances energy expenditure and heat production while reducing fat accumulation. Elevated levels of crotonylation have been found to inhibit the expression of glycerol‐3‐phosphate dehydrogenase 1 and fatty acid binding protein 4, thereby suppressing lipolysis and browning of white adipocytes. Conversely, increased crotonylation promotes the expression of adenylate kinase 2, triosephosphate isomerase 1, and NADH:ubiquinone oxidoreductase subunit A8, which enhances lipolysis and browning of white adipocytes. Modulating the function of target proteins through control of their crotonylation levels presents a promising strategy for obesity treatment.^172^ Furthermore, HDAC1 has been shown to inhibit adipocyte‐mediated thermogenesis by upregulating the crotonylation level of PGC1a/UCP1 in inguinal WAT.^173^

#### 4.7.3 Malonylation in obesity

In the context of obesity, malonylation is linked to the AMPK–acetyl‐CoA carboxylase (ACC) signaling pathway and SIRT5. AMPK phosphorylates ACC1 at Ser79 and ACC2 at Ser212, thereby inhibiting the conversion of acetyl coenzyme A to malonyl coenzyme A.^174^ Malonyl coenzyme A serves as a precursor for fatty acid synthesis and as a malonyl donor in lysine malonylation.^104^ Reduced malonyl coenzyme A levels may impede protein malonylation and attenuate fatty acid synthesis, thereby influencing obesity development. Recent studies have shown that SIRT5 deficiency leads to elevated levels of protein malonylation, reduced glycolysis, and diminished basal mitochondrial respiration in primary chondrocytes.^175^

### 4.8 The cysteine modifications

#### 4.8.1 Palmitoylation in obesity

CD36 molecule (CD36) facilitates fatty acid uptake via a mechanism involving dynamic palmitoylation‐regulated endocytosis. Upon binding of fatty acids to CD36, its downstream kinase LYN is activated. Adenine phosphoribosyltransferase APT1 depalmitoylates CD36, subsequently recruiting another tyrosine kinase, SYK, which promotes the phosphorylation of c‐Jun N‐terminal kinase, thus initiating fatty acid endocytosis. Inhibition of CD36 endocytosis by targeting either LYN or SYK effectively hinders CD36‐dependent lipid droplet growth in adipocytes and attenuates HFD‐induced weight gain in mice.^176^

#### 4.8.2 S‐sulfhydration in obesity

S‐sulfhydration related to H2S is intricately linked with lipid accumulation and catabolism. Acting as a novel adipokine, H2S plays a pivotal role in modulating various metabolic processes including glucose uptake, lipid storage, and mobilization, thereby contributing to the development of obesity. Notably, H2S induces sulfhydration of perilipin 1, a protein intimately involved in lipid droplet dynamics. This sulfhydration event results in reduced phosphorylation of perilipin 1, consequently impeding isoprenaline‐stimulated lipolysis.^177^

### 4.9 Drugs targeting PTM in obesity

#### 4.9.1 β3 Agonist

Mirabegron, a beta3 agonist, has gained approval for treating overactive bladder syndrome. In a recent clinical study, mirabegron was found to induce the expression of beige adipose markers, along with phosphorylation of hormone‐sensitive lipase at serine 660. This effect was observed in obese, insulin‐resistant subjects, leading to the production of beige adipose tissue in the subcutaneous WAT of humans^178^

#### 4.9.2 GLP‐1

Semaglutide, a GLP‐1 agonist, presents a promising option for weight loss therapy in obese patients. In a randomized, double‐blind trial involving 1961 overweight or obese adults with a body mass index ≥ 30 and without diabetes, significant weight reduction was observed in the Semaglutide group compared with the placebo group, with mean weight changes of −15.3 kg (−14.9%) and −2.6 kg (−2.4%), respectively. Notably, nausea and diarrhea were the predominant adverse effects reported.^179^ Furthermore, Semaglutide has been shown to reverse the reduction in serine phosphorylation of calcium voltage‐gated channel subunit alpha1 A, calcium voltage‐gated channel subunit alpha1 B, and calcium voltage‐gated channel subunit alpha1 D proteins induced by HFD in obese mice.^180^ In a 68‐week phase 3b clinical trial, weekly subcutaneous injections of semaglutide demonstrated significantly greater weight loss compared with once‐daily subcutaneous injections of liraglutide, when combined with dietary and physical activity guidance^181^

#### 4.9.3 SIRT1 agonist

SRT3025 administration has been found to elevate hepatic p65 and skeletal muscle Foxo1 deacetylation levels, consequently leading to reductions in plasma LDL cholesterol and total cholesterol (TC) levels, ultimately preventing atherosclerosis in high cholesterol diet‐fed ApoE(−/−) mice.^182^ In a randomized, double‐blind crossover study involving 11 healthy obese men, resveratrol was shown to ameliorate metabolic parameters in obese individuals. Resveratrol's mechanisms involve the activation of citrate synthase through upregulation of SIRT1 and PGC‐1α protein levels in muscle, along with modulation of intracellular lipid levels and inflammatory markers.^183^ Moreover, resveratrol demonstrates efficacy in alleviating diet‐induced obesity in mice by inhibiting PGC‐1α acetylation and subsequently enhancing its activity.^184^

#### 4.9.4 HAT inhibitor

Salt inducible kinase 2 functions by directly phosphorylating p300 at Ser89, leading to inhibition of its HAT activity. Consequently, downregulation of salt inducible kinase 2 expression promotes enhanced lipogenesis and contributes to the development of hepatic steatosis.^185^

#### 4.9.5 Others

IκB kinase promotes phosphorylation of CYLD lysine 63 deubiquitinase to promote ubiquitination of NRF2 and exacerbates oxidative stress injury in obesity‐associated nephropathy, and in vitro experiments have demonstrated that inhibitors of IκB kinase reduce lipid deposition and reactive oxygen species production, among others.^186^

## 5 PROTEIN PTMs IN HYPERLIPIDEMIA

**FIGURE 3:**
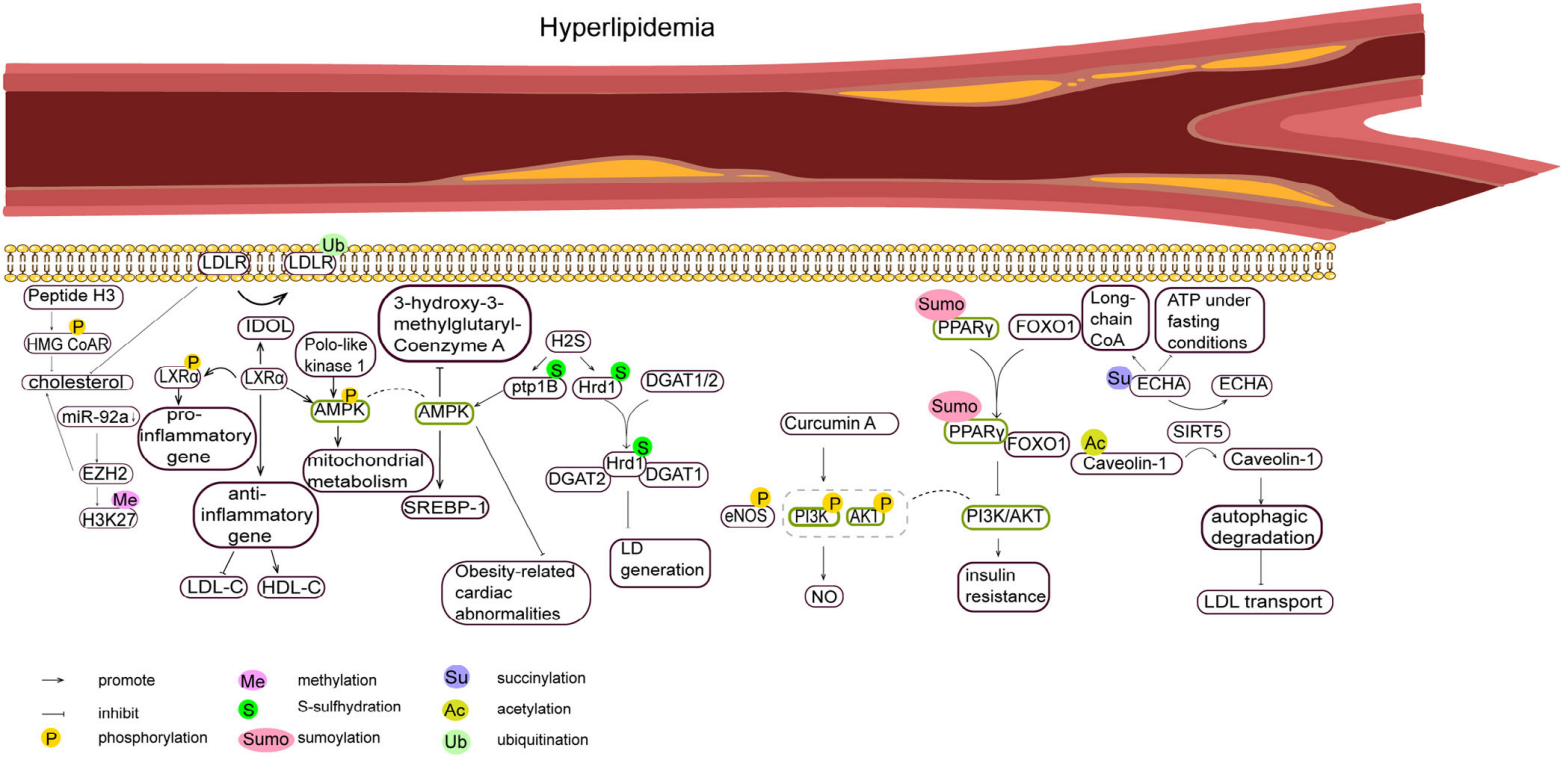
PTMs in hyperlipidemia. Stimulated by internal and external factors, multiple protein posttranslational modifications influence the course of hyperlipidemia. (A)The level of phosphorylation of AMP‐activated protein kinase (AMPK) is regulated by recombinant liver X receptor alpha (LXRα), poly‐like kinase1, and so on, which affects mitochondrial metabolism, and obesity‐related cardiovascular abnormalities. (B) SUMOylation modification of peroxisome proliferator‐activated receptor γ (PPARγ) affects the activity of the phosphatidylinositol 3‐kinase (PI3K)/AKT serine/threonine kinase (AKT) pathway, which in turn leads to insulin resistance, and the level of phosphorylation of PI3K/AKT itself affects the production of NO. (C) Sirtuin 5 (SIRT5) mediates deacetylation of Caveolin‐1 and desuccinylation of hydroxyacyl–CoA dehydrogenase trifunctional multienzyme complex subunit alpha (ECHA), thereby affecting lipid transport and fatty acid oxidation.

With economic development and changes in dietary habits, hyperlipidemia, characterized by high blood lipid levels, is increasingly affecting human health and can lead to a series of more serious diseases such as atherosclerosis and coronary heart disease.^187^ It has been shown that PTMs mediate the onset of hyperlipidemia and related disorders in a variety of ways (Figure 3).

### 5.1 Phosphorylation in hyperlipidemia

Phosphorylation of LXRα and the AMPK pathway are key players in hyperlipidemia. LXRα, a central transcriptional regulator of inflammatory and metabolic genes, exhibits differential effects based on the phosphorylation status of serine 196 (S196). Phosphorylation at S196 promotes an inflammatory response, whereas unphosphorylated LXRα inhibits inflammation. Mice with phosphorylation‐deficient LXRα show reduced levels of LDL cholesterol (LDL‐C) and increased levels of high‐density lipoprotein cholesterol, resulting in a decreased risk of atherosclerosis.^188^ Additionally, phosphorylation of LXRα at S196 has been implicated in promoting atherosclerosis by regulating the macrophage transcriptome.^189^ The physical activity of LXRα induces the biosynthesis of prosolubilized lipid mediators and enhances mitochondrial metabolism through AMPK phosphorylation, thereby offering protection against cardiovascular disease.^190^ AMPK plays a crucial role in phosphorylating SREBP‐1c at Ser372, inhibiting its activity and consequently attenuating atherosclerosis.^191^ Polo‐like kinase 1 stimulates AMPK phosphorylation, thus alleviating atherosclerosis.^192^ Furthermore, peptide H3 has been shown to reduce cholesterol levels in hepatocyte cells by upregulating the phosphorylation of 3‐hydroxy‐3‐methylglutaryl‐coenzyme A reductase and inhibiting its activity through the AMPK pathway.^193^

### 5.2 Ubiquitination in hyperlipidemia

IDOL, also referred to as myosin‐regulated light chain‐interacting protein, possesses the RING structural domain and plays a crucial role in regulating lipid metabolism, thus impacting the development of hyperlipidemia. Acting as an E3‐ubiquitin ligase, IDOL facilitates the direct transfer of ubiquitin from E2 to target proteins. Particularly, IDOL targets the LDL receptor (LDLR) for ubiquitination and subsequent degradation. Mice with IDOL knockout exhibit reduced serum cholesterol levels, decreased body weight, diminished adipose tissue mass, and mitigated liver lipid accumulation.^194^ The regulation of cholesterol uptake via the LDLR pathway is orchestrated by LXR, which transcriptionally induces IDOL expression to prompt ubiquitination of the cytoplasmic structural domain of LDLR.^195^ Mice expressing IDOL variants with inhibited degradation mechanisms, such as IDOL‐antidegraded or dominantly active forms, demonstrate elevated plasma LDL‐C levels and heightened atherosclerotic lesion formation.^196^ Notably, the G51S variant of IDOL disrupts IDOL self‐ubiquitination and enzymatic degradation, leading to stabilized IDOL protein, enhanced LDLR degradation, and increased levels of LDL‐C.^197^

### 5.3 Acetylation in hyperlipidemia

Lipid metabolism is intricately linked to the acetylation of dynamin‐related protein and cyclic AMP‐responsive element‐binding protein 3‐like 3, hepatocyte specific (CREBH). Moreover, the Sirtuin family of deacetylases plays a crucial role in regulating lipid levels.

Excessive lipid accumulation has been associated with increased acetylation of dynamin‐related protein, leading to its activation and subsequent mitochondrial translocation, thereby exacerbating cardiomyocyte dysfunction.^198^ Acetylation of CREBH at lysine 294 enhances its transcriptional activity and is vital for maintaining hepatic lipid homeostasis during fasting. Mutation of lysine 294 in CREBH results in hepatic steatosis and hyperlipidemia in animals subjected to prolonged fasting.^199^

SIRT5 facilitates the deacetylation of Caveolin‐1, a membrane‐intrinsic protein, promoting its autophagic degradation. This process inhibits LDL transport in the presence of Caveolin‐1, thus delaying the progression of atherosclerosis.^200^ Additionally, SIRT6‐mediated deacetylation of histone H3 by FOXO3 promotes a repressive chromatin state, contributing to the regulation of hypercholesterolemia in mice.^201^

### 5.4 Methylation in hyperlipidemia

Histone methylation levels play a pivotal role in the pathogenesis of atherosclerosis. Primarily involving H3K9 and H3K27, alterations in histone methylation are significant in atherosclerotic lesions. Studies have indicated reduced levels of H3K9me2 and H3K27me2 in these lesions, with no observable changes in H3K4me2. However, there is an upregulation in the expression of the histone methyltransferases MLL2 and G9a. Additionally, decreased levels of H3K9me2 in smooth muscle cells and inflammatory cells, as well as reduced dimethylated H3K27 in inflammatory cells, have been observed.^202^ These findings suggest a potential association between histone methylation and the development of atherosclerosis.

Hyperhomocysteinemia (HHcy) stands as an independent risk factor for cardiovascular diseases, including atherosclerosis. MiR‐92a has been identified as a regulator of EZH2 expression in HHcy‐induced atherosclerosis. Overexpression of EZH2 promotes the level of H3K27me3 and leads to the accumulation of triglycerides (TG) and TC. Therefore, targeting miR‐92a may hold promise as a novel therapeutic approach for managing HHcy‐associated atherosclerosis.^203^

### 5.5 Glycosylation in hyperlipidemia

The status of glycosylation is intricately associated with lipid metabolism. Congenital dysglycosylation perturbs both protein and lipid glycosylation processes, along with abnormalities in glycogenes. Transmembrane protein 199 regulates serum levels of LDL‐C, TC, and non‐high‐density lipoprotein cholesterol, thereby influencing the development of hypercholesterolemia. Altered glycosylation status may impact lipoprotein metabolism by enhancing protein degradation and affinity for proatherogenic lipoproteins, reducing secretion, modifying lipid profiles, and influencing correct localization.^204^ Furthermore, the glycosylation profiles of immunoglobulin G were cross‐sectionally correlated with the risk of cardiovascular disease and subclinical atherosclerosis, with immunoglobulin G glycoprotein GP18 exhibiting a highly negative correlation with very LDL.^205^

### 5.6 The lysine modifications in hyperlipidemia

#### 5.6.1 Succinylation in hyperlipidemia

Succinylation also participates in hyperlipidemia. It regulates cardiac function through SIRT5 and impedes fatty acid oxidation by downregulating the activity of hydroxyacyl–CoA dehydrogenase trifunctional multienzyme complex subunit alpha (ECHA), a key protein involved in this process. Hearts from mice lacking SIRT5 exhibit reduced ECHA activity, elevated levels of long‐chain acyl‐CoA, and decreased ATP levels under fasting conditions. Therefore, targeting SIRT5 holds promise for mitigating cellular damage induced by ischemia and reperfusion injury.^206^

#### 5.6.2 Crotonylation in hyperlipidemia

Adenosine extract of Ganoderma lucidum (AEGL) exhibits lipid‐lowering and antiatherogenic properties. In HFD‐induced hyperlipidemic mice, AEGL intervention significantly alters the acetylation and crotonylation of proteins involved in fatty acid metabolism. Specifically, the levels of crotonylation of acyl‐CoA oxidase 1 and acyl‐CoA synthetase long‐chain family member 5 are upregulated, while the levels of crotonylation of fatty acid binding protein 2, acyl‐CoA synthetase long‐chain family member 1, and acyl‐CoA synthetase medium‐chain family member 3 are downregulated following AEGL treatment. These proteins play crucial regulatory roles in peroxisomal oxidation and fatty acid β‐oxidation pathways.^207^

#### 5.6.3 Lactylation in hyperlipidemia

In the context of hyperlipidemia, lactylation plays a significant role. Upon M1 activation, the substantial production of lactate resulting from aerobic glycolysis initiates the endogenous “lactate clock.” Lactylation is linked to genomic alterations in macrophages, driving metabolic reprogramming and promoting the transition of M1‐type macrophages to M2‐type macrophages. This transition induces the expression of genes involved in tissue repair.^208^ Additionally, exercise training has been found to enhance lactylation of methyl‐CpG binding protein 2, thereby inhibiting atherosclerosis. Further investigations have revealed that lactylation of methyl‐CpG binding protein 2 suppresses the expression of extra‐epitope regulatory proteins by binding to chromatin.^209^

#### 5.6.4 Malonylation in hyperlipidemia

Malonylation is implicated in hyperlipidemia, where short‐chain fatty acids such as propionate, malonate, butyrate, 2‐hydroxyisobutyrate, β‐hydroxybutyrate, crotonate, succinate, and glutarate, along with their corresponding acylations (propionylation, malonylation, butyration, 2‐hydroxyisobutyrylation, β‐hydroxybutyrylation, crotonylation, succinylation, and glutarylation), play pivotal roles in CVD.^210^

Studies have revealed that knockdown of FAS and elevated levels of malonyl coenzyme A induce malonylation of mTOR at lysine 1218 (K1218). This malonylation of mTOR K1218 detrimentally affects mTORC1 activity.^211^ Presently, research on the relationship between malonylation and hyperlipidemia predominantly focuses on malonylation‐associated enzymes, with limited exploration into the specific regulatory mechanisms underlying malonylation. Further in‐depth investigation in this area holds promise for the development of targeted therapies for hyperlipidemia.

### 5.7 The cysteine modifications in hyperlipidemia

#### 5.7.1 Palmitoylation in hyperlipidemia

CD36 levels are elevated in atherosclerosis.^212^ DHHC4 and DHHC5 play distinct roles in different subcellular compartments, regulating the palmitoylation, plasma membrane localization, and fatty acid uptake activity of the scavenger receptor CD36. Mice lacking DHHC4 or DHHC5 exhibit reduced fatty acid uptake activity in adipose tissue.^213^

#### 5.7.2 S‐sulfhydration in hyperlipidemia

S‐sulfhydration emerges as a critical player in the context of hyperlipidemia. Within endothelial cells, the catabolism of cysteine and cystathionine leads to the generation of H2S and its associated sulfur compounds (H2Sn) through a process termed the trans‐sulfuration pathway. This pathway assumes particular significance in the vascular system, where cystathionine gamma cleavage enzyme plays a pivotal role in the production of vascular H2S/H2Sn. Notably, GYY4137, a novel slow‐release H2S compound, exerts inhibitory effects on ox‐LDL‐induced lipid accumulation. Furthermore, it mitigates vascular inflammation and oxidative stress, enhances endothelial function, and attenuates the formation of atherosclerotic plaques.^214^ The downregulation of protein tyrosine phosphatase protein tyrosine phosphatase nonreceptor type 1 prevents obesity‐related cardiac abnormalities by activating AMPK.^215^ Conversely, the inhibition of H2S activity, achieved through the induction of protein tyrosine phosphatase nonreceptor type 1 sulfation at Cys 215, serves as a protective mechanism for the cardiovascular system.^216^ Additionally, the S‐sulfation of HMG‐CoA reductase degradation protein 1 (HRD1) mediated by NaHS enhances the interaction of HRD1 with diacylglycerol O‐acyltransferase 1/2, thereby impeding the formation of lipid droplets.^217^

### 5.8 Drugs targeting PTM in hyperlipidemia

#### 5.8.1 Statin

Rosuvastatin, a selective inhibitor of HMG‐CoA reductase, has shown promising outcomes in clinical trials. In a study involving 543 Chinese patients with subclinical atherosclerosis, rosuvastatin significantly attenuated the progression of carotid intima‐media thickness over a span of 2 years, with favorable tolerability.^218^ Moreover, a multicenter, clinically randomized trial conducted in South Korea, which included 1,894 patients, demonstrated that a combination therapy comprising moderate‐intensity rosuvastatin and ezetimibe led to a higher proportion of patients achieving LDL concentrations below 70 mg/dL.^219^ Additionally, rosuvastatin was found to enhance SIRT1 expression and modulate protein acetylation levels, with 12 proteins showing upregulation and 6 proteins exhibiting downregulation out of 100 proteins with detectable acetylation signals.^220^

## 6 PROTEIN PTMs IN NAFLD

**FIGURE 4:**
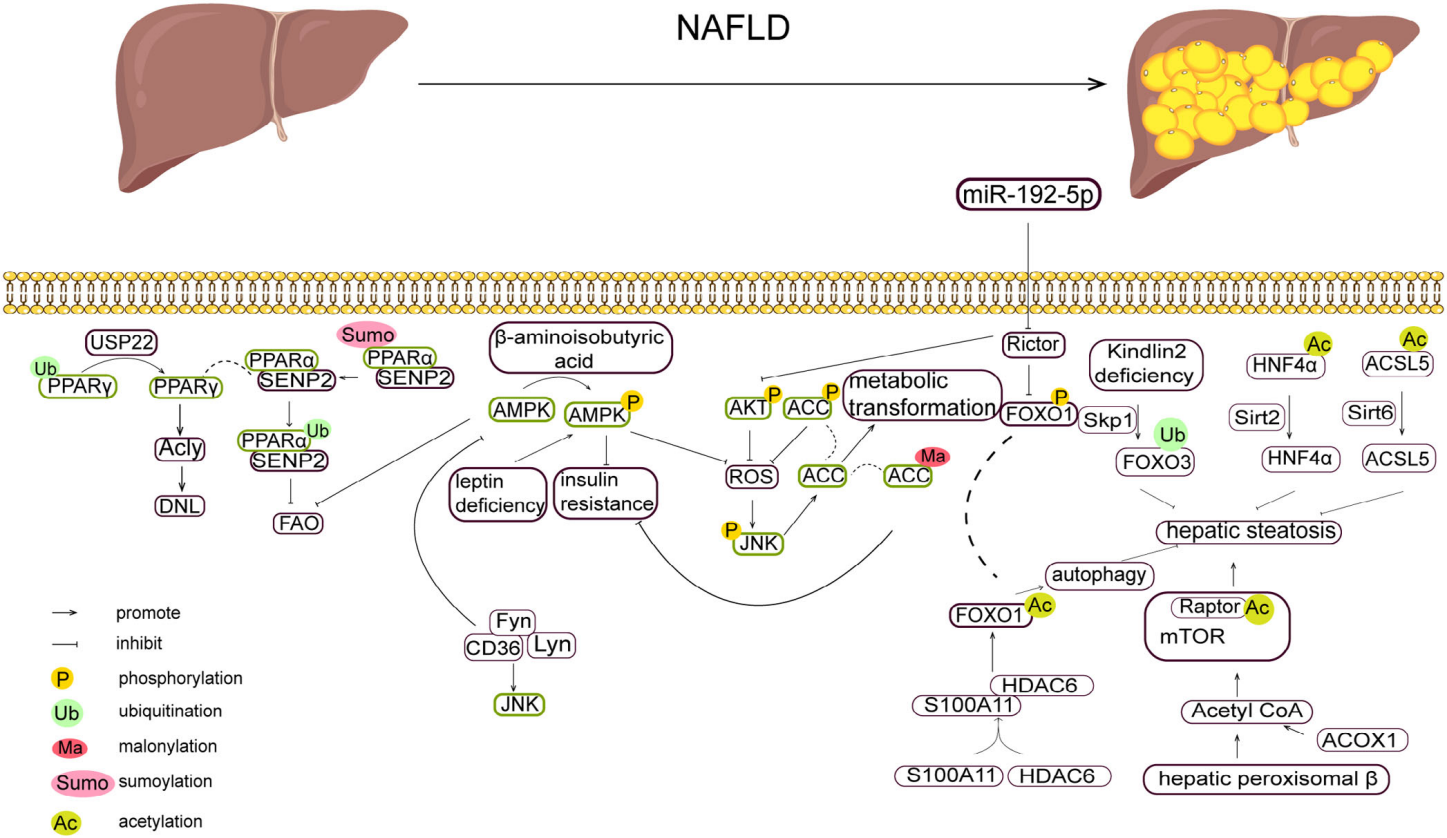
PTMs in NAFLD. Nonalcoholic fatty liver disease (NAFLD) is characterized by excessive hepatic lipid deposition, including simple fatty liver, nonalcoholic steatohepatitis (NASH) and its associated cirrhosis, and a variety of protein modifications are involved in the NAFLD disease process. (A) Peroxisome proliferator‐activated receptor (PPAR) is involved in the process of NAFLD, and ubiquitin specific peptidase 22 (USP22)‐mediated deubiquitylation of PPARγ promotes de novo lipogenesis (DNL), and de SUMOylation inhibited fatty acid oxidation. (B) The phosphorylation level of AMP‐activated protein kinase (AMPK) was regulated by leptin, β‐aminoisobutyric acid, and so on, which affected insulin resistance and reactive oxygen species (ROS) production. (C) Liver steatosis levels are regulated by ubiquitination of Forkhead box O3 (FOXO3), acetylation of Forkhead box O1 (fOXO1), hepatocyte nuclear factor 4α (HNF4α), acyl‐CoA synthetase long‐chain family member 5 (ACSL5), and Raptor.

NAFLD, as one of the most common liver diseases in the world, affects about one‐third of the global population, and its prevalence is increasing year by year, which is seriously threatening the world's public health security. Epigenetic modification of histone proteins has been confirmed in many studies as an important mechanism affecting the development of NAFLD, among others (Figure 4).^221^

### 6.1 Phosphorylation in NAFLD

Protein phosphorylation plays a pivotal role in regulating NAFLD. Elevated levels of serine/threonine kinase 17b lead to reduced phosphorylation of serine/arginine‐rich splicing factor 6 thereby influencing the alternative splicing of genes related to mitochondrial function and exacerbating lipid accumulation. Pharmacological intervention targeting serine/threonine kinase 17b has shown promising results in ameliorating hepatic steatosis and nonalcoholic steatohepatitis (NASH).^222^ Nicotine exposure has been implicated in the progression of NAFLD to NASH by promoting the phosphorylation of sphingomyelin phosphodiesterase 3 via AMPKα in the ileum, thereby increasing intestinal ceramide formation.^223^ Leptin deficiency in pigs enhances fatty acid β‐oxidation by downregulating JAK2–STAT3 and AMPK phosphorylation, which triggers mitochondrial autophagy and oxidative stress in hepatocytes, contributing to the development of NASH.^224^ AMPK‐mediated phosphorylation of caspase‐6 has been associated with liver damage in NASH, with phosphorylation inhibiting caspase‐6 activity.^225^ Additionally, β‐aminoisobutyric acid has been shown to exert anti‐inflammatory and anti‐insulin resistance effects by inducing AMPK phosphorylation.^226^

Inhibition of ACC hinders the metabolic shifts essential for inducing glycolysis and oxidative phosphorylation during hepatic stellate cell activation.^227^ Mutations in the phosphorylation sites of ACC1 and ACC2 promote hepatic de novo lipogenesis and liver lesions in mice.^228^ The bioactive peptide leucine–glutamine–proline attenuates the severity of NASH in mice by upregulating AMPK and ACC.^229^

Zinc supplementation and strength exercise have been shown to increase the phosphorylation level of AKT at Ser473, thereby improving insulin signaling and mitigating NAFLD in rats with T2DM.^230^ Hepatocyte‐derived exosome miR‐192‐5p induces the activation of macrophage M1‐type polarization by inhibiting mammalian rapamycin target protein Rictor, downregulating the phosphorylation levels of AKT and FOXO1 and activating FOXO1.^231^ The long noncoding RNA LncARSR promotes lipid accumulation by activating the IRS‐2/AKT pathway through inhibiting the phosphorylation level of Yes1 associated transcriptional regulator, whereas silencing lncARSR alleviates NAFLD in mice.^232^ Oxyberberine, a gut microbiota‐mediated oxidative metabolite of berberine, inhibits the aberrant phosphorylation of IRS‐1 and promotes phosphorylation levels of its downstream, thereby improving insulin signaling to alleviate NAFLD.^233^

### 6.2 Ubiquitination in NAFLD

Hepatic steatosis is intricately associated with the ubiquitination of ATP citrate lyase (ACLY), lipid droplet‐encapsulated protein, and Kindlin‐2‐related proteins.

ACLY undergoes ubiquitination and subsequent degradation mediated by HRD1, thereby inhibiting its lipogenic effects. Overexpression of ACLY in db/db mice led to a reduction in the severity of NAFLD and diminished steatosis in isolated liver progenitor cells.^234^ Ubiquitin‐specific protease 22 stabilizes PPARγ by deubiquitination in hepatocellular carcinoma, consequently upregulating ACLY expression and promoting de novo lipogenesis.^235^

The essential dietary amino acids leucine and isoamino acid bind strongly to the ubiquitin protein ligase E3 component recognin 1, facilitating the ubiquitination of lipid droplet‐encapsulated protein perilipin 2 and counteracting hepatic steatosis. These findings were corroborated in studies involving Drosophila melanogaster cells and hepatocyte‐like cells.^236^ Additionally, during adipocyte differentiation, perilipin 2 is degraded via the ubiquitin–proteasome pathway.^237^

Kindlin‐2 indirectly impacts NAFLD by modulating the ubiquitination of associated proteins. Kindlin‐2 deficiency inhibits hepatic steatosis by promoting Skp1‐dependent ubiquitination and proteasomal degradation of the transcription factor FOXO2. Mice with hepatocyte‐specific Kindlin‐2 deficiency exhibited enhanced weight gain, reduced abnormal accumulation of hepatic lipid droplets, lower serum levels of TG, TC, and LDL, and markedly improved hepatic function.^238^ Furthermore, Kindlin‐2 deficiency facilitates the ubiquitination and degradation of the antioxidant glutathione S‐transferase P1 in hepatocytes, leading to oxidative stress and inflammatory factors in hepatocytes.^239^

### 6.3 Acetylation in NAFLD

Protein acetylation plays a significant role in the pathogenesis of NAFLD by influencing hepatic fatty acid oxidation, lipogenesis, and lactate accumulation.

NAFLD is characterized by the excessive accumulation of lipids in the liver, a process regulated by various genes. Cytoplasmic SIRT6 exerts its effect by deacetylating acyl‐CoA synthetase long‐chain family member 5, thereby promoting fatty acid oxidation and reducing lipid accumulation.^240^ SIRT2 also plays a role in NAFLD by deacetylating HNF4α, delaying the onset of hepatic steatosis.^241^ Additionally, a high‐fat diet has been shown to upregulate the expression of S100 calcium‐binding protein A11, which, in turn, promotes adipogenesis and lipid accumulation by enhancing the acetylation of the transcription factor FOXO1 and activating autophagy and adipogenic processes.^242^ The pathological damage resulting from excessive lipid uptake, known as lipotoxicity, is a crucial step in the progression from simple steatosis to NASH.^243^ Studies in rodents, pigs, and rhesus monkeys have demonstrated that arachidonate 12‐lipoxygenase inhibits the lysosomal degradation of ACC1, a pivotal enzyme involved in the conversion of acetyl coenzyme A to malonyl coenzyme A. Reduced arachidonate 12‐lipoxygenase activity leads to decreased ACC1 expression and lower levels of malonyl coenzyme A, resulting in the accumulation of acetyl coenzyme A.^244^

Lactate accumulation stands out as a notable feature in the progression of NAFLD. The clearance of lactate, a crucial process, is mediated by lactate dehydrogenase B (LDHB) and plays a significant role in influencing NAFLD advancement. Studies have revealed that the lysine residue at position 82 of LDHB is acetylated by P300/CBP‐associated factor, resulting in LDHB inactivation. This acetylation event significantly impairs hepatic lactate clearance, leading to lactate accumulation and exacerbating the progression of NAFLD.^245^

Moreover, hyperuricemia has been implicated in promoting hepatic fat accumulation and the upregulation of lipogenic genes, thereby contributing to NAFLD development. It has been demonstrated that hyperuricemia activates c‐Jun N‐terminal kinase, consequently increasing the expression of lipogenic enzymes such as FAS and acetyl‐CoA carboxylase 1, both in vivo and in vitro.^246^ Additionally, a study suggests that acetyl‐CoA generated from the oxidation of hepatic peroxisomal β inhibits autophagy and promotes hepatic steatosis through the activation of mTORC1. The enzyme acyl‐CoA oxidase 1, which is involved in hepatic lipid metabolism, regulates the cellular membrane acetyl‐CoA content. This, in turn, mediates the acetylation of Raptor, a component of mTORC1, and its lysosomal localization, ultimately leading to the activation of mTORC1, a key regulator of autophagy.^247^ Furthermore, the E3 ligase SH3 structural domain‐containing ring finger 2 has been identified as an inhibitor of cell membrane acetyl‐CoA production. It promotes ubiquitin‐dependent degradation, resulting in reduced synthesis and deposition of de novo lipids and cholesterol.^248^

### 6.4 Methylation in NAFLD

Protein methylation levels intricately regulate liver tissue regeneration and hepatic metabolism. Histone methylation plays a pivotal role in promoting the regeneration of damaged liver tissue. Research by Wang et al.^249^ suggests that a decrease in DNA methylation triggers the redistribution of repressive histone methylation, such as H3K27me3, thereby enhancing the regeneration process in liver tissues. The knockout of brain‐specific oxidation resistance 1a stimulates the accumulation of H3K2me2, which in turn modulates the production of growth hormone in the pituitary gland. Growth hormone is known to exert significant effects on hepatic metabolism and the development of fatty liver. Male mice lacking oxidation resistance 1a display a propensity for developing fatty liver, a condition further corroborated by the concurrent reduction in intrahepatic growth hormone levels.^250^ Moreover, HFD is associated with increased expression of retinoic acid‐inducible gene‐I, which promotes cholesterol synthesis and steatosis, thereby inducing NASH. The demethylase Jumonji domain containing 4 counters this process by demethylating retinoic acid‐inducible gene‐I, leading to its reduced expression and consequently inhibiting the progression of NASH.^251^

### 6.5 SUMOylation in NAFLD

SUMOylation levels within the farnesoid X receptor (FXR), and PPARα pathways exhibit distinct effects on the progression of NAFLD. SUMO specific peptidase 1 catalyzes the de‐SUMOylation of receptor‐interacting protein kinase 1. This action inhibits receptor‐interacting protein kinase 1‐driven cell death and inflammatory activation, thereby delaying the development of NAFLD.^252^ Additionally, mice lacking SENP2 demonstrate resistance to hepatic steatosis and obesity induced by a high‐fat diet. SENP2 interacts with PPARα, promoting its de‐SUMOylation, ubiquitination, and subsequent degradation. Consequently, this inhibits fibroblast growth factor 21 expression and fatty acid oxidation.^253^

Furthermore, FXR agonists have exhibited therapeutic potential in the treatment of NASH and liver fibrosis. However, recent research has revealed that activation of hepatic stellate cells can upregulate FXR SUMOylation, thereby inhibiting FXR signaling. This attenuation diminishes the efficacy of FXR agonists. Consequently, the combination of SUMOylation inhibitors with FXR agonists has emerged as a promising strategy against hepatic fibrosis.^254^

### 6.6 Glycosylation in NAFLD

The glycosylation levels of proteins undergo significant alterations in NAFLD. Specifically, in NAFLD, binding bead protein and transferrin glycosylation sites become hyperglycosylated, whereas these glycosylation sites are differentially occupied under physiological conditions. Serum protein hyperglycosylation is anticipated to serve as a biomarker for the early stages of NAFLD.^255^ Furthermore, loss of glycosylation of key uptake and efflux transporter proteins upon progression to NASH affects the function of transporter proteins.^256^ NASH is associated with increased expression of mannose, complex/fucoidan glycosylated, and heterogeneous N‐glycan structures, and the spatial distribution of free N‐glycan abundance correlates with NASH severity.^257^

CREBH undergoes covalent modification by N‐acetylglucosamine, and deglycosylation of CREBH inhibits its hydrolysis. The addition of unglycosylated CREBH promotes lipogenesis, induces expression of lipotoxicity and inflammation, and exacerbates liver injury in mice with NAFLD. Moreover, overexpression of alpha‐1,6‐mannosylglycoprotein 6‐beta‐N‐acetylglucosaminyltransferase promotes N‐glycosylation of CREBH, which ameliorates liver lesions by enhancing its transcriptional activity and affecting the activities of PPARα and stearoyl‐CoA desaturase‐1.^258^

### 6.7 The lysine modifications in NAFLD

#### 6.7.1 Succinylation in NAFLD

Alterations in metabolism‐related protein succinylation levels have been observed in NAFLD. A comprehensive quantitative analysis of the succinylome in rat livers with NAFLD revealed 243 succinylation sites on 178 proteins exhibiting altered succinylation levels. These succinylated proteins were primarily localized in the cytoplasm and mitochondria, and they were implicated in a diverse array of metabolic pathways and cellular processes. These pathways include carbon metabolism, amino acid metabolism, fatty acid metabolism, as well as functions related to binding, catalysis, antioxidant defense, and xenobiotic metabolism.^259^

#### 6.7.2 Crotonylation in NAFLD

Crotonylation also plays a significant role in NAFLD, particularly in liver fibrosis. Liver fibrosis, a critical step in the progression of NAFLD to cirrhosis, involves the replacement of healthy liver tissue with fibrotic tissue and regenerative nodules due to chronic inflammation.^260^ Spearman's correlation analysis reveals a negative correlation between the degree of lysine crotonylation and serum fibrosis indicators. Sorafenib, a multikinase inhibitor known for its antihepatic fibrosis effects, is shown to reverse the crotonylation of H2BK12 and H3K18, suggesting that lysine crotonylation mediates the antifibrotic effects of sorafenib.^261^

#### 6.7.3 Lactylation in NAFLD

In the context of NAFLD, protein lactylation influences hepatic lipid deposition. Elevated levels of mitochondrial pyruvate carrier 1 (MPC1) positively correlate with hepatic lipid accumulation in NAFLD. However, hepatic lipid accumulation is attenuated in MPC1‐knockout mice. Mechanistically, MPC1 knockout modulates lactate levels in hepatocytes, leading to the downregulation of lactylation at K673 of FAS, thereby inhibiting FAS activity and reducing hepatic lipid accumulation.^262^

#### 6.7.4 Malonylation in NAFLD

Malonylated ACC1 plays a pivotal role in regulating NAFLD progression. Treatment with ML355 effectively reduces ACC1 abundance, malonyl coenzyme A production, and protein malonylation in the livers of mice. Additionally, the metabolite IMA‐1, derived from ML355, exhibits high levels and stability in plasma, serving as the active metabolite of ML355. IMA‐1 treatment inhibits lipid accumulation, enhances fatty acid β‐oxidation, reduces ACC1 levels, blocks protein malonylation, and suppresses hepatocyte cell death.^263^ Furthermore, an intermittent ketogenic diet alleviates steatosis and insulin resistance in NAFLD by downregulating hepatic lysine malonylation of ACC1.^264^

### 6.8 The cysteine modifications in NAFLD

#### 6.8.1 Palmitoylation in NAFLD

Palmitoylation of CD36 contributes to the progression of NASH. CD36 expression increases during the transition from normal liver to simple steatosis and further to NASH. Notably, the distribution of CD36 on the plasma membrane of hepatocytes significantly increases in NASH patients, mirroring findings from mouse experiments. Palmitoylation of CD36 facilitates its translocation to the plasma membrane of hepatocytes in NASH, enhancing the binding and uptake of long‐chain fatty acids and promoting the formation of CD36/Lyn complexes. An imbalance between fatty acid uptake and oxidation, along with activation of inflammatory responses, contributes to the development of NASH.^265^ Exploring inhibitors of CD36 palmitoylation may represent a promising avenue for NASH treatment.

#### 6.8.2 S‐sulfhydration in NAFLD

S‐sulfhydration of unc‐51 like kinase 1, FXR, and KEAP1 emerges as pivotal regulators of hepatic lipid deposition and inflammation. The transcription factor SREBP‐1c plays a central role in inducing hepatic lipid accumulation by upregulating lipid synthesis and concurrently downregulating autophagy, thus impairing lipid degradation.^266^ Notably, SREBP‐1c exerts inhibitory effects on unc‐51 like kinase 1 persulfation, consequently disrupting autophagic lipolytic metabolism, and promoting the development of fatty liver. This dysregulation is facilitated by alterations in H2S levels orchestrated by SREBP‐1c, which in turn downregulates cystathionine γ‐lyase expression through miR‐216a, further exacerbating hepatic lipid accumulation.^267^ Furthermore, endogenous hepatic cystathionine gamma cleavage enzyme/H2S promotes the thiolation of FXR at the Cys138/141 locus, consequently regulating lipid and glucose metabolism and exerting protective effects against inflammation, fibrosis, and NASH.^268^ Additionally, H2S mediates the S‐sulfhydration of KEAP1, activating NRF2 signaling and thereby attenuating HFD‐induced hepatic lipid deposition and liver injury.^269^

### 6.9 Drugs targeting PTM in NAFLD

#### 6.9.1 FXR agonist

The FXR serves as a pivotal player in various pathogenic pathways associated with NASH, including bile acid synthesis, enterohepatic circulation, lipid and glucose metabolism, inflammation, fibrosis, intestinal barrier integrity, and intestinal microbiota regulation. Its activation by bile acids has positioned FXR as one of the most promising targets for current NASH treatments.^270^ FXR is susceptible to PTMs, including phosphorylation, SUMOylation, and acetylation, which can modulate its activity.^271^ Animal experiments have shown that enhanced SUMOylation of FXR strongly impairs FXR signaling and that SUMOylation inhibitors can enhance the efficacy of FXR agonists to delay the progression of liver fibrosis.^254^

Cilofexor (GS‐9674), a small molecule nonsteroidal agonist that acetylates FXR, has emerged as a potential therapeutic agent. In a double‐blind, placebo‐controlled, phase 2 trial involving 140 noncirrhotic NASH patients, cilofexor demonstrated efficacy in reducing hepatic steatosis, improving liver biochemistry, and enhancing serum bile acids levels.^272^

#### 6.9.2 ACC inhibitor

Firsocostat (GS‐0976), a liver‐targeted ACC inhibitor, promotes ACC phosphorylation and subsequent activation. In a phase 2 clinical trial comprising 126 NASH and fibrosis patients, Firsocostat demonstrated efficacy in inhibiting de novo lipogenesis and reducing hepatic steatosis.^273^ Curcumin, alternatively, has been found to inhibit the O‐GlcNAc signaling pathway, thereby reducing hepatic lipid accumulation and inflammation. In NAFLD mice, curcumin also enhances antioxidant responses, thus mitigating the condition.^274^ A randomized, double‐blind, placebo‐controlled trial involving 77 subjects corroborated these findings, showing that curcumin significantly decreased liver fat content and improved serum lipid and transaminase levels in NAFLD patients.^275^

#### 6.9.3 SGLT‐2 inhibitor

Empagliflozin, an SGLT‐2 inhibitor, triggers autophagy activation by enhancing AMPK phosphorylation. This action results in decreased mTOR levels and increased microtubule associated protein 1 light‐chain 3 beta expression, effectively slowing down the progression of NAFLD.^276^

#### 6.9.4 AMPK agonist

Ginsenoside Rb2 demonstrates significant improvement in glucose tolerance in male db/db mice while concurrently restoring hepatic autophagy function. This restoration leads to a reduction in hepatic lipid accumulation, thereby ameliorating NAFLD by reversing the inhibition of the autophagic pathway induced by elevated levels of fatty acids and glucose.^277^ Additionally, Rb2 phosphorylates AKT, thereby enhancing insulin sensitivity in mice with diet‐induced obesity.^278^ Furthermore, triptolide induces hepatic ACC1 phosphorylation by activating AMPK, consequently ameliorating hepatic lipogenesis, fatty acid oxidation, and fibrosis in NAFLD.^279^

#### 6.9.5 HDAC inhibitor

Givinostat demonstrated efficacy in mitigating hepatic fibrosis induced by methionine–choline‐deficient diet in NASH mice, accompanied by a notable suppression of inflammation‐related gene expression. Additionally, Givinostat exhibited a significant reduction in lipid accumulation induced by palmitic acid, thus showcasing its potential as a promising therapeutic agent for human NASH treatment.^280^ Moreover, Givinostat exerts its antifibrotic effects and inhibits hepatic stellate cell proliferation by upregulating the acetylation level of superoxide dismutase 2.^281^

#### 6.9.6 Others

Animal experiments demonstrated that the de‐O‐GlcNAcylase OGA inhibitor Thiamet‐G alleviated APAP‐induced liver injury by inhibiting GSH supplementation.^282^

## 7 DISCUSSION

Metabolism‐related diseases such as diabetes, obesity, hyperlipidemia, and NAFLD present escalating challenges to human well‐being. The main metabolism‐related PTMs include phosphorylation, ubiquitination, acetylation, methylation, palmitoylation, succinylation, crotonylation, lactylation, malonylation, sumoylation, S‐sulfhydration, glycosylation, and others, offer novel avenues and strategies for addressing the prevention and treatment of these conditions. AMPK, PPAR and their upstream and downstream proteins are inhibited or activated through PTM, which in turn affects lipid metabolism and mediates the development of hyperlipidemia. PTM can mediate NAFLD development by affecting oxidative stress, lactate accumulation, autophagy, insulin resistance, and inflammation, and the major signaling pathways affected include PPAR, AMPK, AKT, and JAK2–STAT3. In obesity, PTM affects cellular autophagy, leukolipid browning, lipogenesis and metabolism mediated by signaling pathways such as AMPK, PPAR, and AKT (Table 1).

**TABLE 1:** PTMs and their targets in metabolic diseases.

PTM	DM‐related targets of PTM	Obesity‐related targets of PTM	Hyperlipidemia ‐related targets of PTM	NAFLD‐related targets of PTM	References
Phosphorylation	AMPK, GLUT1, IRS1, AKT, IRS, endothelial nitric oxide synthase, mitsugumin 53	SIRT1, AMPK	LXRα, AMPK, HMGCoAR, AKT, eNOS	SRSF6, JAK2–STAT3, AMPK, ACC, AKT	^52^, ^222^, ^224^
Acetylation	HNF‐4α, neuronal differentiation 1, FOXO3a, thioredoxin interacting protein, PGC1β, LXRα, FOXO1	PRDM16	DRP1, CREBH, Caveolin‐1	Acyl‐CoA synthetase long‐chain family member 5, HNF4α, FOXO1, LDHB, RAPTOR (a component of mTORC1)	^73^, ^82^, ^200^, ^247^
Methylation	NF‐κB,	JAK2	–	Retinoic acid‐inducible gene‐I	^159^, ^251^
Ubiquitination	p27	AKT	LDLR	ACLY, PLIN2	^64^, ^234^, ^236^
Palmitoylatio	GLUT4, Mucin 2	CD36	CD36	FOXO2	^107^, ^176^
Succinylation	–	UCP1	–	–	
Crotonylation	GLUT3	–	ACOX1, ACSL5, FABP2, ACSL1, ACSM3	–	^82^, ^207^
Lactation	–	–	MECP2	FAS	^209^
Malonylation	–	–	mTOR	ACC1	^211^
Sumoylation	GAPDH, XBP1s, Tomosyn1	ERp44, C/EBPβ, SETDB1	PPARγ1	RIPK1, FXR	^163^, ^165^, ^252^
S‐sulfhydration	SIRT1, KEAP1	pLIN1	PTP1B	ULK1, FXR, KEAP1	^215^, ^266^
Glycosylation	AGEs	RAGE	PMM2, ALG6, B4GALT1, CCDC115, TMEM199	Binding bead protein, transferrin, Mac‐2‐binding protein, CREBH, AGEs	^204^, ^258^

PTM plays a pivotal role in the pathogenesis of metabolism‐related diseases and offers innovative avenues for clinical intervention. Developing agonists or inhibitors targeting PTM has emerged as a prominent focus in drug development for these conditions (Table 2).

**TABLE 2:** Drugs targeting PTM in metabolic diseases.

Metabolic diseases	Targets	Drugs	References
Diabetes	SGLT‐2 inhibitor	Ipragliflozin	
	GLP1R agonist	Exendin 4, liraglutide	^117^
	GCK agonist	Dorzagliatin	^119^
	SIRT1 activator	Resveratrol	^124^
	HDAC inhibitor	NaB, HC toxin	^128^
Hyperlipidemia	HHMG‐CoA reductase inhibitor	Rosuvastatin	^220^
Obesity	β3‐adrenergic receptor agonist	Mirabegron	^178^
	GLP‐1	Semaglutide	^179^
	SIRT1 agonist	SRT3025, resveratrol	^182^
	HAT inhibitor	SIK2	^185^
NAFLD	FXR agonist	Cilofexor	^272^
	ACC inhibitor	Firsocostat, curcumin	^273^
	SGLT‐2 inhibitor	Empagliflozin	^276^
	AMPK agonist	Ginsenoside Rb2, triptolide	^277^
	HDAC inhibitor	Givinostat	^280^

Despite substantial advancements in understanding the relationship between PTMs of proteins and metabolic diseases, several deficiencies and limitations persist. The diversity and complexity of PTMs, as well as the mechanisms by which they influence metabolic diseases, remain incompletely elucidated. Furthermore, the translation of preclinical findings to clinical applications is inefficient, the development of targeted interventional drugs is lagging, and research into novel PTMs is still in its infancy and requires urgent, in‐depth exploration.

To advance PTM‐based treatments for metabolism‐related diseases, several avenues warrant exploration: First, establishing a comprehensive data management platform to integrate existing research findings on PTM and metabolism‐related diseases is imperative. This unified platform will facilitate comprehensive analysis and interpretation of available data. Second, further research is essential to elucidate the mechanistic relationship between PTM and metabolism‐related diseases. Leveraging animal studies and clinical trials, we can delve deeper into understanding the underlying mechanisms. This approach will enable the identification of potential PTM targets for the prevention and treatment of metabolic diseases.

## AUTHOR CONTRIBUTIONS

*Design, and supervision; revising of original draft*: Yanqi Dang. *Design, and supervision; manuscript editing and revision*: Guang Ji. *Writing of original draft*: Yunuo Yang. *Manuscript editing and revision*: Jiaxuan Wu and Wenjun Zhou. The final manuscript has been reviewed and approved by all the authors.

## CONFLICT OF INTEREST STATEMENT

All the authors declare no conflict of interest.

## ETHICS STATEMENT

Not applicable.

## Data Availability

Not applicable.
